# The Design, Synthesis, and Biological Activities of Pyrrole-Based Carboxamides: The Novel Tubulin Inhibitors Targeting the Colchicine-Binding Site

**DOI:** 10.3390/molecules26195780

**Published:** 2021-09-24

**Authors:** Sergei Boichuk, Aigul Galembikova, Kirill Syuzov, Pavel Dunaev, Firuza Bikinieva, Aida Aukhadieva, Svetlana Zykova, Nazim Igidov, Ksenia Gankova, Maria Novikova, Pavel Kopnin

**Affiliations:** 1 Department of Pathology, Kazan State Medical University, 420012 Kazan, Russia; ailuk000@mail.ru (A.G.); grop2019@gmail.com (K.S.); dunaevpavel@mail.ru (P.D.); firuza1995@mail.ru (F.B.); arom1705@mail.ru (A.A.); 2 Central Research Laboratory, Kazan State Medical University, 420012 Kazan, Russia; 3 Perm State Academy of Pharmacy, 614990 Perm, Russia; zykova.sv@rambler.ru (S.Z.); igidov_nazim@mail.ru (N.I.); gankova.kseniya@mail.ru (K.G.); 4 Cytogenetics Laboratory, Carcinogenesis Institute, N.N. Blokhin National Medical Research Center of Oncology, 115478 Moscow, Russia; mvnovikova94@mail.ru (M.N.); pbkopnin@mail.ru (P.K.)

**Keywords:** microtubules, tubulin depolymerization, cell cycle, mitotic arrest, apoptosis, breast, lung, prostate cancer, paclitaxel, vinblastine, 2-amino pyrroles

## Abstract

Microtubule targeting agents (MTAs) that interfere with the dynamic state of the mitotic spindle are well-known and effective chemotherapeutic agents. These agents interrupt the microtubule network via polymerization or depolymerization, halting the cell cycle progression and leading to apoptosis. We report two novel pyrrole-based carboxamides (CAs) (CA-61 and -84) as the compounds exhibiting potent anti-cancer properties against a broad spectrum of epithelial cancer cell lines, including breast, lung, and prostate cancer. The anti-cancer activity of CAs is due to their ability to interfere with the microtubules network and inhibit tubulin polymerization. Molecular docking demonstrated an efficient binding between these ligands and the colchicine-binding site on the tubulin. CA-61 formed two hydrogen bond interactions with THR 179 (B) and THR 353 (B), whereas two hydrogen bonds with LYS 254 (B) and 1 with ASN 101 (A) were identified for CA-84. The binding energy for CA-84 and CA-61 was −9.910 kcal/mol and −9.390 kcal/mol. A tubulin polymerization assay revealed a strong inhibition of tubulin polymerization induced by CA-61 and -84. The immunofluorescence data revealed the disruption of the tubulin assembly in CA-treated cancer cells. As an outcome of the tubulin inhibition, these compounds halted the cell cycle progression in the G2/M phase, leading to the accumulation of the mitotic cells, and further induced apoptosis. Lastly, the in vivo study indicated that CAs significantly inhibited the HCC1806 breast cancer xenograft tumor growth in a nude mouse model. Collectively, we identified the novel CAs as potent MTAs, inhibiting tubulin polymerization via binding to the colchicine-binding site, disrupting the microtubule network, and exhibiting potent pro-apoptotic activities against the epithelial cancer cell lines both in vitro and in vivo.

## 1. Introduction

The microtubules are important regulators of a broad spectrum of cellular processes including cell proliferation and migration, vesicle trafficking during endocytosis, and chromosomal segregation during mitosis. The last process is considered as an attractive molecular target for anti-cancer drugs, which leads to the development of numerous natural and synthetic small-molecule compounds, interfering with the microtubule network and inducing the apoptotic cell death of cancer cells due to the mitotic catastrophe [1,2]. The molecular mode of action of microtubule targeting agents (MTAs) is due to their ability to interfere with microtubule polymerization and dynamics via the inhibition or enhancement of tubulin polymerization. The agents that inhibit tubulin polymerization are known as the “microtubule-destabilizing” agents (MDA) (e.g., vinca alkaloids, colchicine, and its analogs), and inhibit the microtubule network via binding to specific sites (i.e., domains) in tubulin. The vinca alkaloids bind to a site of beta-tubulin located in the interface between tubulin dimers [3], whereas colchicine binds to a site of beta-tubulin located at the intra-dimer interface between the alpha- and beta-subunits [4]. In addition to these drugs, two other agents (maytansine and pironetin) have recently been discovered as the potent MDAs inhibiting tubulin polymerization via binding to the domains that are distinct from the vinca alkaloid- and colchicine-binding sites [5,6]. The agents promoting tubulin assembly and regulating microtubules stability are named “microtubule-stabilizing” agents (MSAs) (e.g., taxol, docetaxel). These MSAs bind to the “taxoid” domain in β-tubulin [7,8]. Aside from taxanes, the marine sponge products (e.g., laulimalide [9] and peloruside A [10]) also enhance microtubule polymerization and stabilize the microtubules via binding to the non-taxane domain on β-tubulin [11].

In addition to the “classical” chemotherapeutic agents selectively interfering with the microtubule’s dynamic state, several kinase inhibitors were recently shown to interfere with the tubulin polymerization. For example, Tivantinib was initially developed as a non-ATP competitive inhibitor of c-met and entered a clinical trial as a targeted drug [12]. However, subsequent experiments also illustrated its pro-apoptotic activities against c-met-addicted and non-addicted cancer cells [13]. The experiments performed as a follow-up of this unexplainable notice identified the unexpected activity of Tivantinib to inhibit microtubule polymerization due to its direct binding to the colchicine-binding site on the tubulin [14]. Another targeted drug, Rigosertib, that was originally identified as a polo-like kinase 1 (Plk1) inhibitor [15] was recently shown as a potent microtubule depolymerizing agent, exhibiting its cytotoxic properties for cancer cells via tubulin depolymerization as a consequence of its binding to the tubulin [16], thereby highlighting the possibility of developing anticancer drugs with a dual-mode of action and effectively inhibiting kinase activity and microtubule function. Indeed, the binding sites of colchicine and taxanes are large in size, fairly deep, and mainly hydrophobic [7], properties which are similar to those of the catalytic site of kinases. Strikingly, nocodazole, a well-known microtubule depolymerizing agent, was found as a high-affinity ligand of several cancer-related kinases, including ABL, c-KIT, BRAF, and MEK [17,18]. The detailed mechanisms illustrating the dual mode of action of the kinase inhibitors, which exhibited tubulin depolymerizing potencies, were recently reviewed by *Tanabe K*. [19]. Conversely, several kinase inhibitors, that were clinically approved or currently assayed in phase 2 or phase 3 clinical trials (e.g., selonsertib, masitinib, nintedanib, PF0477736, SNS-314 mesylate, MPI0479605, and ponatinib) were recently found to be potent MSAs without a direct interaction with tubulin [20], thereby suggesting that tubulin itself could be an off-target inhibitor of some kinase inhibitors. Thus, the microtubule-stabilizing effects of kinase inhibitors may account for their therapeutic and adverse side effects, as well.

MTAs (primarily taxanes and vinca alkaloids) are commonly administered to patients with a broad spectrum of solid tumors and hematological malignancies, and highly effective when used in combination chemotherapeutic settings at the initial period of chemotherapy. However, the extended use of MTAs is limited due to the acquired resistance of tumors to these drugs. This might be due to the distinct molecular mechanisms, including the overexpression of ABC-transporters, such as P-glycoprotein in tumor cells [21,22], the altered expression of specific β-tubulin isotypes [23], mutations in β-tubulin, the delay in G2/M transition, and the impairment of the mitotic checkpoints and apoptotic pathways [24]. Moreover, MTA can induce serious adverse effects, including bone marrow toxicity, immunosuppression, and neuropathy, which limits their long-term use and remains a driving force for the development of the novel potent and less-toxic drugs, which effectively interfere with the microtubules network.

We previously reported the potent anti-cancer activities of ethyl-2-amino-pyrrole-3-carboxylates (EAPCs) in vitro and in vivo [25,26,27]. The molecular mechanism of action was due to their ability to interfere with the microtubule dynamic state by inhibiting the tubulin polymerization, the induction of the cell cycle arrest in the M-phase, and apoptotic cell death.

The other findings also illustrate the potency of pyrrole-based compounds to behave in a similar way to the microtubule polymerization-targeting agents, leading to cell cycle arrest and apoptosis in cancer cells. In 2017, Carta et al. showed the anti-proliferative activity of novel phenylpyrroloquinolinones (PPyQs) against the leukemic and solid tumor cell lines [28], and the best effect was achieved by a compound which indicated a similar binding mode for the known inhibitor plinabulin, a first-in-class selective immunomodulating microtubule-binding agent that bound in the vicinity of the colchicine-binding domain of β-tubulin in αβ-tubulin heterodimers. In 2019, Brindisi et al. illustrated the cell cycle and inhibitory and pro-apoptotic effects of a new series of pyrrolonaphthoxazepines [29]. In our efforts to discover novel tubulin inhibitors, we developed novel pyrrole-based analogs targeting the colchicine-binding site on tubulin and thereby interfering with tubulin polymerization. By utilizing a multi-step screening procedure based on both computational and biological methods, we selected two compounds (CA-61 and -84) exhibiting the highest negative binding energy value in the colchicine-binding domain of tubulin. Biological studies revealed that these compounds effectively inhibited tubulin polymerization and disrupted the microtubule network. As an outcome of these effects, CAs induced a robust cell cycle arrest in the G2/M phase, and the apoptosis of the epithelial cancer cell lines, including breast, lung, and prostate cancer. Importantly, these compounds exhibited potent anti-tumor activities in mouse xenograft models, thereby illustrating a discovery of the novel and effective compounds exhibiting anti-cancer properties via targeting the microtubules.

## 2. Results

### 2.1. Synthesis of 2-Aminopyrrole Derivatives

**Scheme 1:**
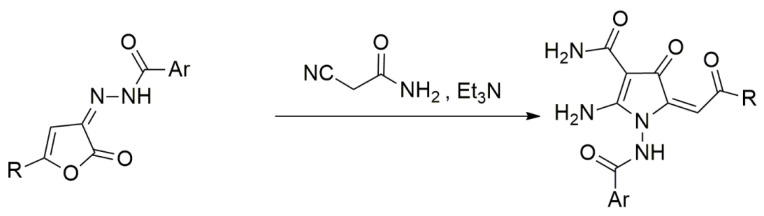
The common synthetic pathway of the derivatives of 2-aminopyrroles.

Given that the reactions of N-substituted 3-imino(hydrazono)furan-2(3H)-ones with CH-nucleophiles in the presence of triethylamine led to the formation of 2-aminopyrrole derivatives exhibiting potent anticancer activities in vitro and in vivo [25,26,27,30,31,32], we synthesized the novel aminopyrrole derivatives. The synthesis of the compounds was carried out according to Scheme 1. The synthesis of the compounds was carried out as follows: 3.63 g (0.01 mol) of furanone I and 1.13 g (0.01 mol) of cyanoacetic acid ethyl ester were dissolved by heating in 80 mL of anhydrous toluene, 1 g (0.01 mol) of triethylamine was added and boiled for 30 min, and then the mixture was cooled. The resulting colorless precipitate was filtered off and recrystallized from ethanol. The progress of the reactions and the purity of the synthesized compounds were monitored by TLC on Silufol plates (developed with iodine vapor) in the diethyl ether—benzene—acetone 10:9:1 system. Based on the thin-layer chromatography (TLC) data of the compounds and their spectral characteristics, the purity of the compounds was more than 99%.

### 2.2. Cytotoxic Activities of 2-Aminopyrroles in Epithelial Cancer Cell Lines

We initially examined the cytotoxic activities of the synthesized pyrrole-based compounds against the epithelial cancer cell lines, including breast and lung cancer. The cellular viability of cancer cells treated with the compounds was examined by an MTS-based assay and IC_50_ values were calculated. We found that 13 compounds, that were most effective against all cancer cell lines, were used in the present study. These compounds exhibited cytotoxic activities against epithelial cancer cell lines at a concentration of <25 μM. The representative IC_50_ values of pyrrole-based compounds in MDA-MB-231 breast cancer cell lines are shown in Supplementary Table S1. The similar cytotoxic activities of these compounds were observed in HCC1806 breast and H1299 lung cancer cell lines.

### 2.3. Molecular Docking of Pyrrole-Based Analogs Selected the Compounds Which Effectively Bound to a Colchicine-Binding Site in Tubulin

Next, we performed the computational analysis of the most active compounds and utilized molecular docking as a well-known and powerful approach for structure-based drug discovery used to examine the potential ligand–protein interactions [33,34]. Molecular docking was performed using Schrodinger molecular modeling software and the Glide Docking SP and XP modes to assess the compounds’ binding interactions with the tubulin. The tubulin structure (no. 4O2B) was downloaded from the Protein Data Bank (4O2B. Available online: https://www.rcsb.org/structure/4O2B, accessed on 18 August 2021). and prepared for molecular analysis by using the Protein Preparation Wizard module. We initially performed a “blind docking” without assigning a specific binding site to determine the possible protein–ligand interactions. This allowed us to determine the colchicine-binding site as the most feasible dock for the vast majority of CAs. Next, we examined various ligand poses and selected the minimal Glide dock scores as the most feasible binding pose of the ligands. All non-covalent interactions were also analyzed. Lastly, we rescored the docking results obtained by XP Glide by using the Prime/MM-GBSA tool in Maestro, which allowed us to assess the binding efficiency in the implicit water model. For the internal control, avanbulin (also known as BAL27862) was included in the molecular docking analysis and demonstrated the similar interactions with the tubulin observed previously by X-ray crystallography [35]. The Glide SP, XP, MM-GBSA and ligand strain energy values, as well as the description of ligand–protein interactions of the most active compounds are shown in Table 1.

**Figure 1:**
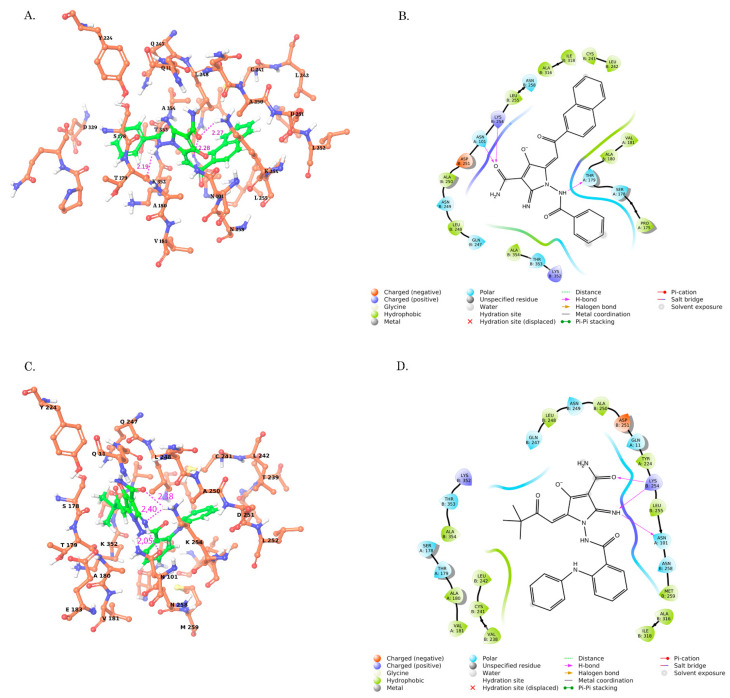
Computer modeling of the CAs binding site on tubulin. (**A**,**C**) 3D diagram illustrating the proposed binding mode of the interactions of CA-61 (**A**) and CA-84 (**C**), with the tubulin (**B**,**D**) 2D Ligand interaction diagrams of the CA-61 (**B**) and CA-84 (**D**) with significant amino acid residues.

Based on the molecular docking data, we found that the two most potent cytotoxic 2-aminopyrroles exhibited the highest tubulin-binding capacities: 2-amino-1-benzamido-5-(2-(naphthylen-2-yl)-2-oxoethylidene)-4-oxo-4,5-dihydro-1-*H*-pyrrole-3-carboxamide (named below as CA-61) and 2-amino-5-(3,3-dimethyl-2-oxobutylidene)-4-oxo-1-(2-phenylamino)-benzamido)-4,5-dihydro-1-*H*-pyrrole-3-carboxamide (named below as CA-84). The proposed binding mode of the interactions of CA-61 and CA-84 with the colchicine-binding site of the tubulin is shown in Figure 1. As shown in Figure 1, CA-84 formed 2 hydrogen bonds with LYS 254 (B) and one with ASN 101 (A), whereas CA-61 formed hydrogen bonds with ASN 101 (A), LYS 254 (B) and THR 179 (B). Importantly, CA-84 and CA-61 exhibited the lowest ligand strain energy values, thereby illustrating the minimal energy required for the adaptation to the receptor-bound conformation (Table 1).

### 2.4. Structure–Activity Relationship (SAR) Analysis of CAs

To determine the active sites of the CAs responsible for their anti-cancer activities, we performed the structure–activity relationship (SAR) analysis of the top-ranking compounds exhibiting the cytotoxic activities against the MDA-MB-231 breast cancer cell line, as was shown in Supplementary Table S1.

Based on the SAR data, we found that the amino acid residues and functional groups provided the most potent cytotoxic activities of CAs. For example, the cytotoxic activity of the compounds presented in Supplementary Table S2, Group I, was solely dependent on the presence of the amino group (shown as R1 in Supplementary Table S2), which is known to increase the binding affinity of the ligand and thereby enhances its potency as a drug molecule. Indeed, the lack of this group in CA-1519I and CA-1573I abrogated their cytotoxic properties, whereas CA-61 was found as a top-ranking cytotoxic compound. Surprisingly, R2 and R3 radicals (naphthalen-2-yl and pyridin-4-yl, respectively) of these compounds did not contribute to their cytotoxic activities. As was mentioned before, the hydrogen bonds with ASN 101 (A), LYS 254 (B), and THR 179 (B) might also have an impact on binding to the target protein (e.g., tubulin) and the cytotoxic activity of CA-61.

**Figure 2:**
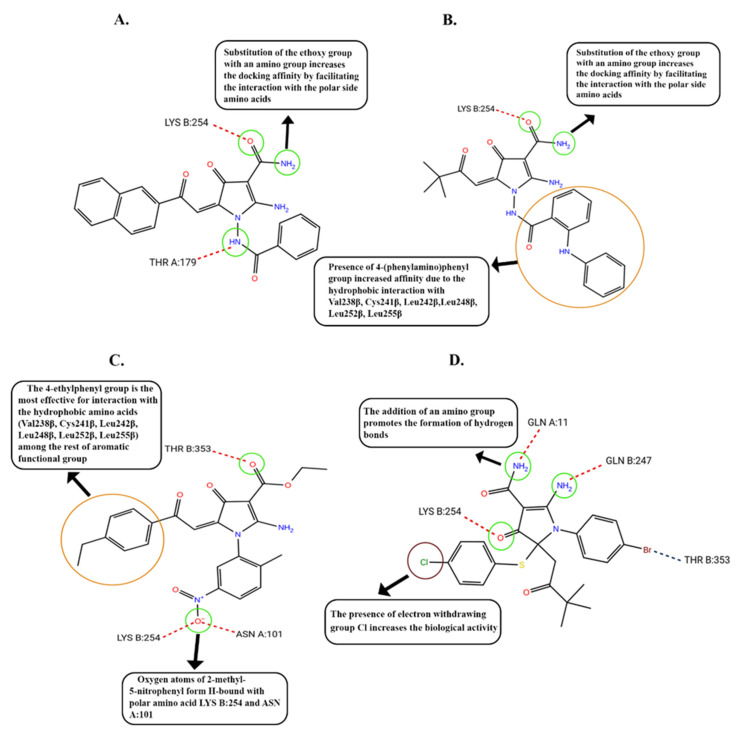
The structures of CA-61 (**A**), CA-84 (**B**), CA-166 (**C**) and CA-1489I (**D**). Amino acid residues and functional groups, responsible for highest biological activities are highlighted in the circles.

Next, the compounds included in Group II in Supplementary Table S2 differed from each other by two radicals. Again, an amino group (shown as R1) played a pivotal role in the cytotoxic activity of CA-84, whereas the other compounds exhibited a similar structural core, but lacked the amino group in position R1 and failed to kill cancer cells in vitro (e.g., CA-1348I and CA-610I). The cytotoxic properties of CA-84 might be also attributed to 4-(phenylamino)phenyl group (shown as R2), circled in Figure 2B and shown in Supplementary Table S2 and Group II. In addition, CA-84 formed two hydrogen bonds with LYS 254 (B) and one with ASN 101 (A), thereby increasing the tubulin-binding properties of this compound, which also might have an impact on its biological activity.

The cytotoxic activities of the compounds included in Group III in Supplementary Table S2 were predominantly attributed to the amino group in the R1 position (this was present only in CA-1489I, exhibiting the maximal cytotoxic activity), whereas CA-1488I and CA-1474I lacked this group in the same position. In addition to the amino group in R1 position, the highest cytotoxic activities of CA-1488I when compared to CA-1474I might be due to the presence of Cl in the R2 position which was attributed to the increase in cytotoxic activity ~ 3-fold.

Finally, all seven compounds included in Group IV exhibited high and moderate cytotoxic properties against the epithelial cancer cell lines (Supplementary Table S2, Group IV). Among them, the most potent were CA-166 and CA-59. This could be attributed to the presence of 4-ethylphenyl in the R1 position, as shown for CA-166. The replacement of C_2_H_5_ by Cl slightly attenuated the cytotoxic activity of CA-59 (Supplementary Table S2, Group IV). At the same time, the replacement of C_2_H_5_ by the CH_3_ induced a 6-fold decrease in the cytotoxic activity of CA-208, whereas the presence of 4-methoxyphenyl in the same position decreased the cytotoxic activity of CAs almost 10-fold (e.g., CA-68).

The spectra characteristics of the compounds used in present study are shown in Supplementary Figure S4. These include the compounds exhibiting high and moderate cytotoxic activities against MDA-MB-231 breast cancer cell lines (i.e., IC50 values ~50 μM), as well as non-active compounds.

Based on the IC_50_ values of the amino-pyrrole derivates (Supplementary Table S1) and the results of their computer modeling (Figure 1 and Figure 2, Table 1 and Supplementary Table S2), we selected two of the top-ranking compounds for further research and examined in detail their biological activities. The synthesis of these compounds, their chemical structures, and their main characteristics are shown below.

### 2.5. Synthesis, Spectrum Measurements and Basic Characteristics of CA-61 and -84

**Figure 3:**
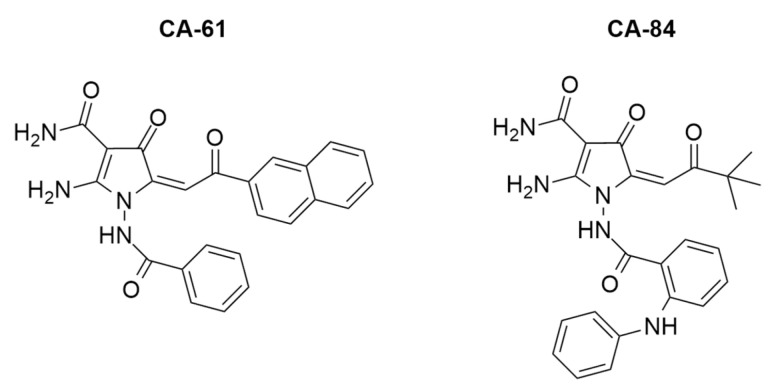
The chemical structures of 2-amino-1-benzamido-5-(2-(naphthylin-2-yl)-2-oxoethylidene)-4-oxo-4,5-dihydro-1-H-pyrrole-3-carboxamide—CA-61 and 2-amino-5-(3,3-dimethyl-2-oxobutylidene)-4-oxo-1-(2-phenylamino)benzamido)-4,5-dihydro-1-H-pyrrole-3-carboxamide—CA-84.

The structures of the most active compounds (i.e., CA-61 and -84) are shown in Figure 3.

These compounds were obtained by the recycling of hydrazones 2,3-furandiones CA-61-a, and CA-84-a under the action of cyanoacetamide in absolute toluene or dioxane, and in the presence of the main catalyst triethylamine (Supplementary Scheme S1).

The compounds were colorless (CA-61), or yellow-colored (CA-84), crystalline substances, soluble in dimethyl sulfoxide (DMSO), dimethylformamide, hardly soluble in ethanol and insoluble in benzene, water and hexane.

The IR spectrum of compound CA-61 was characterized by the presence of absorption bands with stretching vibrations of the NH-groups at 3181–3482 cm^−1^, carbonyl groups at 1666 cm^−1^, as well as stretching vibrations of the double bond C=C at 1615 cm^−1^.

In the ^1^H NMR spectrum of compound CA-61, along with the singlet of the methine proton of the exoethylene bond at 6.85 ppm, a multiplet of aromatic protons was found at 7.59 ppm; the signals of the protons of NH groups were in the range 8.41–10.91 ppm.

The mass spectrum of compound CA-61 contained peaks of molecular and fragment ions with the following *m*/*z* (I,%) values: 426 [M]^+^ (24.0), 349 [M-C_6_H_5_]^+^ (12.0), 271 [M-naphthalene-2-ylCO]^+^ (18.0), 155 [naphthalen-2-ylCO]^+^ (49.0), 105 [C_6_H_5_CO]^+^ (100.0), 77 [C_6_H_5_]^+^ (23.0), which also confirmed the proposed structure.

The IR spectrum of compound CA-84 contained absorption bands of stretching vibrations of the NH group in the range of 3450–3310 cm^−1^, bands of stretching vibrations of the carbonyl groups C=O in the range of 1685–1647 cm^−1^, as well as a double bond at 1598 cm^−1^.

In the ^1^H NMR spectrum of compound CA-84, there was a singlet of nine protons of the tert-butyl radical at 1.14 ppm, a singlet of a methine proton at 6.46 ppm, a multiplet of aromatic protons at 7.37 ppm, and there were proton signals amino groups at 7.11–7.94 ppm.

The mass spectrum of compound CA-84 revealed peaks with the following m/z (I,%) values: 447 (100.0) [M]^+^, 196 (74.0) [2C_6_H_5_NHC_6_H_4_CO)]^+^, 168 (5.5) [2C_6_H_5_NHC_6_H_4_)]^+^.

**Figure 4:**
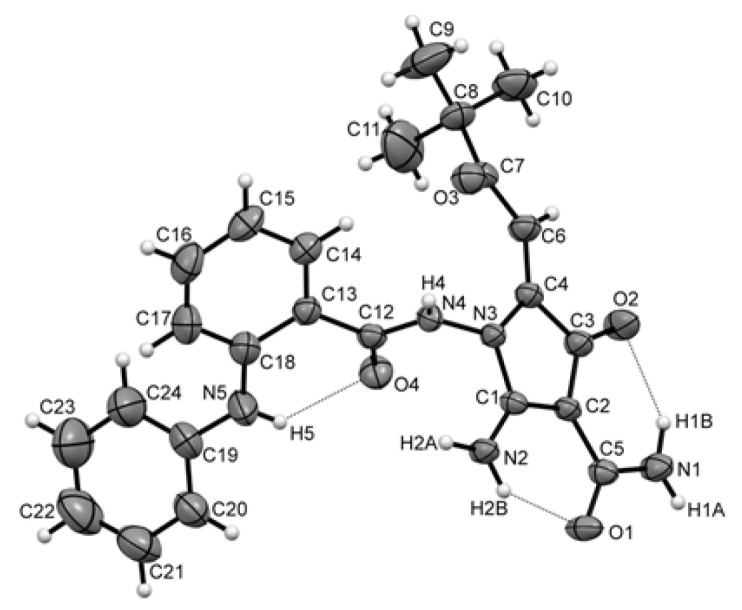
The structure of CA-84 according to X-ray diffraction data in thermal ellipsoids of 50% probability.

To establish the spatial structure of 2-aminopyrroles, we obtained a single crystal of compound CA-84 and performed its X-ray diffraction analysis (Figure 4). The spectral data and physicochemical properties of the CA-84 compound completely coincided with the data of the previously obtained 2-aminopyrrole [36].

Thus, the analysis of free-binding energies using various scoring functions, ligand strain energies, changes in ligand positions, and protein–ligand interactions illustrated that CA-84 was a more stable compound on the colchicine-binding site when compared to CA-61. These two compounds were further subjected for their biological activities in vitro and in vivo.

### 2.6. CAs Inhibit Tubulin Polymerization and Disrupt the Microtubule Network

**Figure 5:**
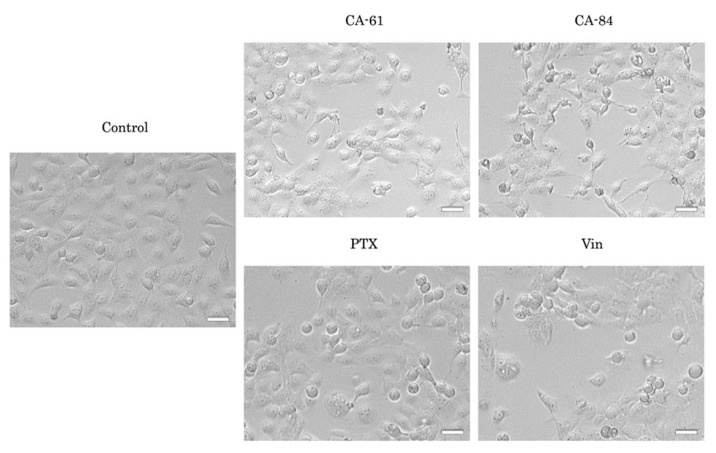
CAs induce changes in the morphology of cancer cells. Morphological changes in HCC1806 breast cancer cells treated with solvent (DMSO) (negative control), CA-61 and -84 (10 μM), Paclitaxel (PTX) (0.5 μM), and Vinblastine (Vin) (0.1 μM). The chemotherapeutic agents were used as a positive control. Cells were treated with CAs and chemotherapeutic agents (indicated above) for 9 h and subjected to light microscopy (Leica). Magnification 10×, scale bars 100 μM.

**Figure 6:**
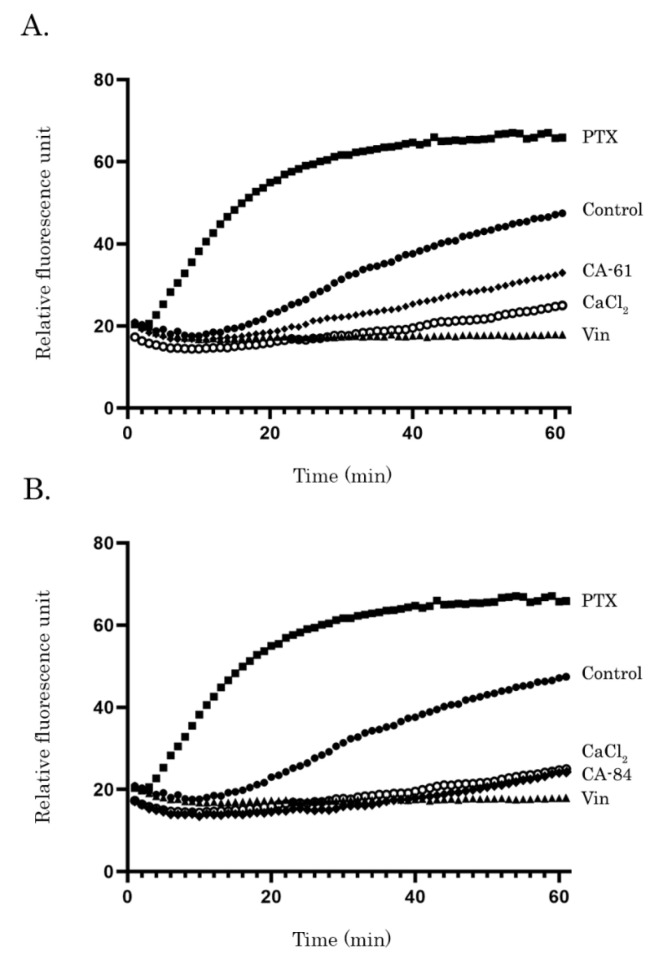
Dynamics of tubulin polymerization in samples treated with CA-61 (**A**) and -84 (**B**). Tubulin was also incubated with DMSO (control), Paclitaxel (PTX), Vinblastine (Vin), and CaCl_2_ at 37 °C, and absorbance was assessed every min for 1 h. A shift of the curve to the upper left of the control (DMSO) represents an increase in polymerized microtubules. A shift to the bottom right of the graph reflects the decrease in the rate of tubulin polymerization.

Given that the epithelial cancer cells lines incubated with CA-61 and -84 acquired a round-shaped morphology, which was similar to the cells treated with Vinblastine (Vin) and paclitaxel (PTX) (Figure 5), and taking into account their high affinity to the colchicine-binding site of tubulin, we sought to examine whether CAs interfered with tubulin polymerization. For this purpose, we performed a cell-free in vitro tubulin polymerization assay based on the ability of dissolved tubulin to polymerize in vitro at 37 °C in the presence of GTP. As expected, this process was substantially accelerated when PTX (a well-known microtubule-stabilizing agent) was mixed with tubulin (Figure 6). Conversely, vincristine (the microtubule depolymerizing agent) effectively inhibited tubulin polymerization (Figure 6). Interestingly, CA-61 and -84 effectively inhibited tubulin polymerization (Figure 6A,B, respectively).

**Figure 7:**
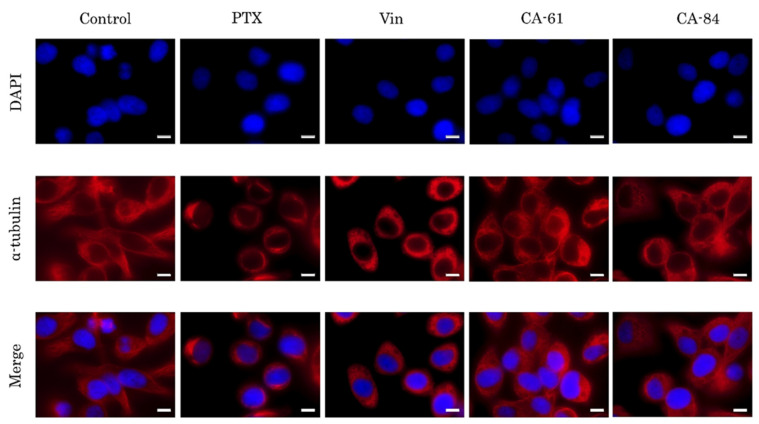
CAs disrupt the microtubules network in cancer cells. HCC1806 breast cancer cells were treated with DMSO (control), Paclitaxel (PTX-0.5 μM), Vinblastine (Vin—0.01 μM), CA-61 and -84 (10 μM) for 9 h, fixed with a mixture of methanol and acetone, and stained with α-tubulin (red). DAPI nuclear staining (blue) was used to outline an equal number of cells. Images were captured by using a fluorescence Olympus BX63 microscope. Magnification 100×, scale bars 10 μM.

The effects of CA-61 and -84 on the tubulin cytoskeleton were also analyzed at a cellular level. For this purpose, HCC1806 breast cancer cells were treated with PTX, Vin, or CAs for 9 h and subjected to immunofluorescence staining to examine their α-tubulin state. The microtubule network was visualized by immunofluorescence microscopy; α-tubulin were stained in red and cellular nuclei were stained in blue with DAPI (4′,6-diamidino-2-phenylindole). We found that both of the CAs effectively disrupted the microtubule network of HCC1806 breast cancer cells (Figure 7), which was evidenced by the diffuse gauze staining of α-tubulin in CA-treated cells when compared to the hairy-like α-tubulin pattern in non-treated (control) cells. Of note, Vin-treated cells exhibited a similar staining pattern for α-tubulin. As expected, the characteristic microtubule bundles were detected in PTX-treated cells, thereby revealing the PTX-induced stabilization of the microtubules after 9 h of treatment (Figure 7).

**Figure 8:**
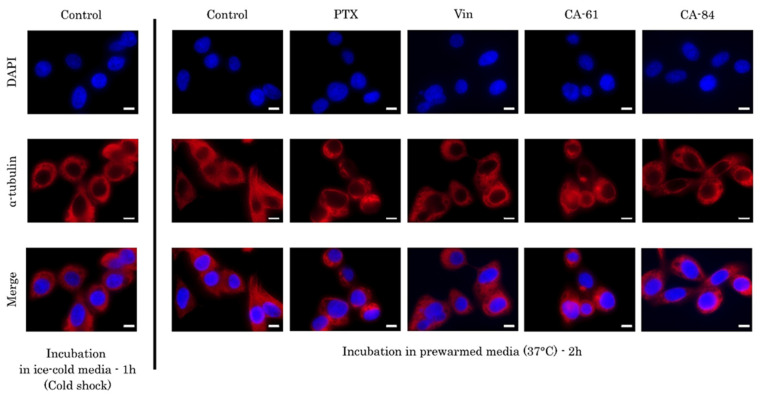
CAs inhibit the microtubules assembly in cold-shocked cancer cells. HCC1806 were incubated in ice-cold media (cold shock) within 1h in presence of DMSO (control), Paclitaxel (PTX—0.5 μM), Vinblastine (Vin—0.01 μM), CA-61 and -84 (10 μM). After 1 h, the media were replaced with prewarmed media (37 °C) and cells were cultured for 2 h to initiate tubulin polymerization. Cells were fixed with a mixture of methanol and acetone, and stained with α-tubulin (red). DAPI nuclear staining (blue) was used to outline an equal number of cells. Images were captured by using a fluorescence Olympus BX63 microscope. Magnification 100×, scale bars 10 μM.

To corroborate these findings, we also performed a depolymerization of the microtubules using cold shock (the cell plates were put on ice during the treatment with CAs for 1 h) and further assessed the dynamics of microtubules polymerization by replacing the ice-cold medium with a pre-warmed culture medium for 37 °C for 2 h to initiate tubulin polymerization. As expected, tubulin polymerization was observed in non-treated control cells (Figure 8), whereas in Vin-treated cells the microtubules network remained disrupted (Figure 8). Strikingly, CAs-treated cells exhibited an α-tubulin staining pattern, which was similar to Vin-treated cells (Figure 8), thereby revealing the tubulin depolymerization properties of CAs.

Collectively, these data indicated that the anti-proliferative potency of CA-61 and -84 was mediated through the inhibition of tubulin polymerization and the disruption of the microtubules network.

### 2.7. CAs Effectively Reduce the Viability of the Epithelial Cancer Cell Lines In Vitro

To examine the cytotoxic activities of CAs against epithelial cancer cell lines, we performed the MTS-based survival assay by using HCC1806 and MDA-MB-231 breast cancer, H1299 non-small cell lung cancer (NSCLC), and PC-3 prostate cancer cell lines. Non-transformed BJ tert fibroblasts were also included in this particular set of experiments. Each cell line was treated with various concentrations of CA-61 and -84 (0.01–100 μM) for 48 h. PTX and Vin were also included as positive controls and exhibited cytotoxic activities in nanomolar concentrations for all cancer cell lines indicated above (e.g., IC_50_ values for PTX varied from 9.6 to 110 nM for the MDA-MB-231 breast and H1299 lung cancer cell lines, respectively). The IC_50_ values for CAs are shown in Table 2.

### 2.8. CA-61 and -84 Induce Cell Cycle Abnormalities and Enhance the Number of Cancer Cells in the G2/M Phase

**Figure 9:**
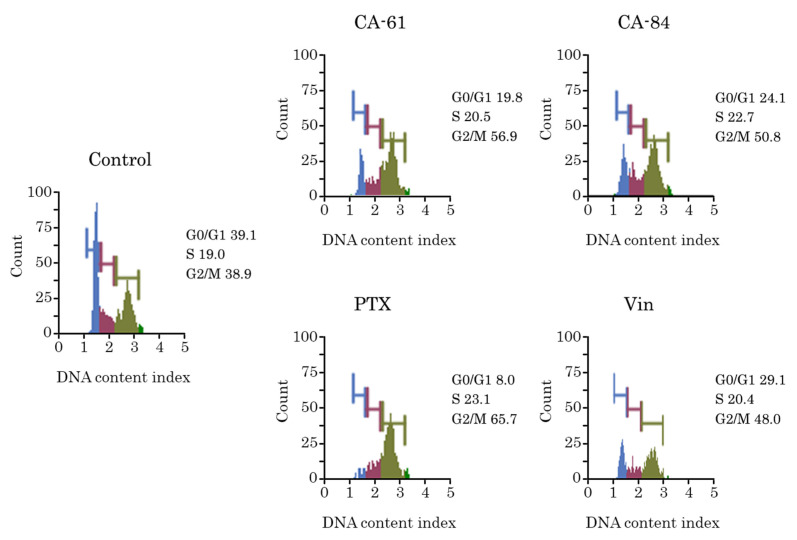
Cell cycle profile of HCC1806 breast cancer cells treated with CA-61, 84, Paclitaxel (PTX), or Vinblastine (Vin). Representative histograms illustrating the increased numbers of cells in the G2/M phase after the treatment with CA-61 and -84 for 9 h. PTX and Vin were used for the positive controls.

Based on the round-shaped morphological changes observed in CA-61 and CA-84-treated epithelial cancer cell lines (Figure 5), we proposed that the growth-inhibitory properties of these compounds might be due to their ability to interfere with the cell cycle regulation and induce the selective accumulation of cancer cells in M-phase. To test this hypothesis, we initially performed an FACs-based cell cycle analysis which revealed the accumulation of breast, lung, and prostate cancer cell lines in G2/M-phase after CA treatment (Figure 9 and Table 3).

### 2.9. CAs Induce Selective Accumulation of Cancer Cells in the M-Phase

**Figure 10:**
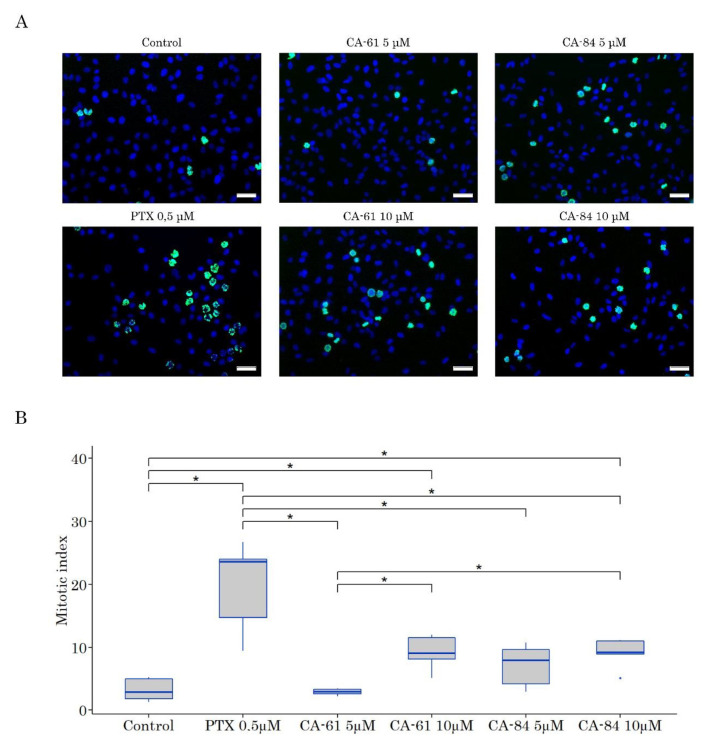
Accumulation of tumor cells in M-phase induced by CAs. (**A**) The representative images (20×) of HCC1806 cells immunostained by using Alexa488-conjugated anti-phospho-histone 3 (Ser10) antibody after incubation with DMSO (negative control), CA-61 (5 and 10 μM), CA-84 (5 and 10 μM) and Paclitaxel (PTX) (0.5 μM, positive control). Cell nuclei were outlined by staining with DAPI. Magnification 20×, scale bars 50 μM. (**B**) The plot representing the mitotic index calculated from 5 low-power fields for each experimental condition indicated above; * *p* < 0.05.

To further corroborate our findings illustrating the CAs ability to induce the arrest of cancer cells in the G2/M phases (Figure 9), we performed immunofluorescence staining to examine the expression of Histone 3 phosphorylated at Ser10 residues (pH3Ser10), a well-known marker of mitotic cells. Indeed, we observed a significant increase in the number of pH3Ser10-positive cells after the CA treatment of HCC1806 breast cancer cells, when compared to the non-treated cells (Figure 10). As expected, PTX also induced a robust increase in mitotic cells in HCC1806 cell lines.

**Figure 11:**
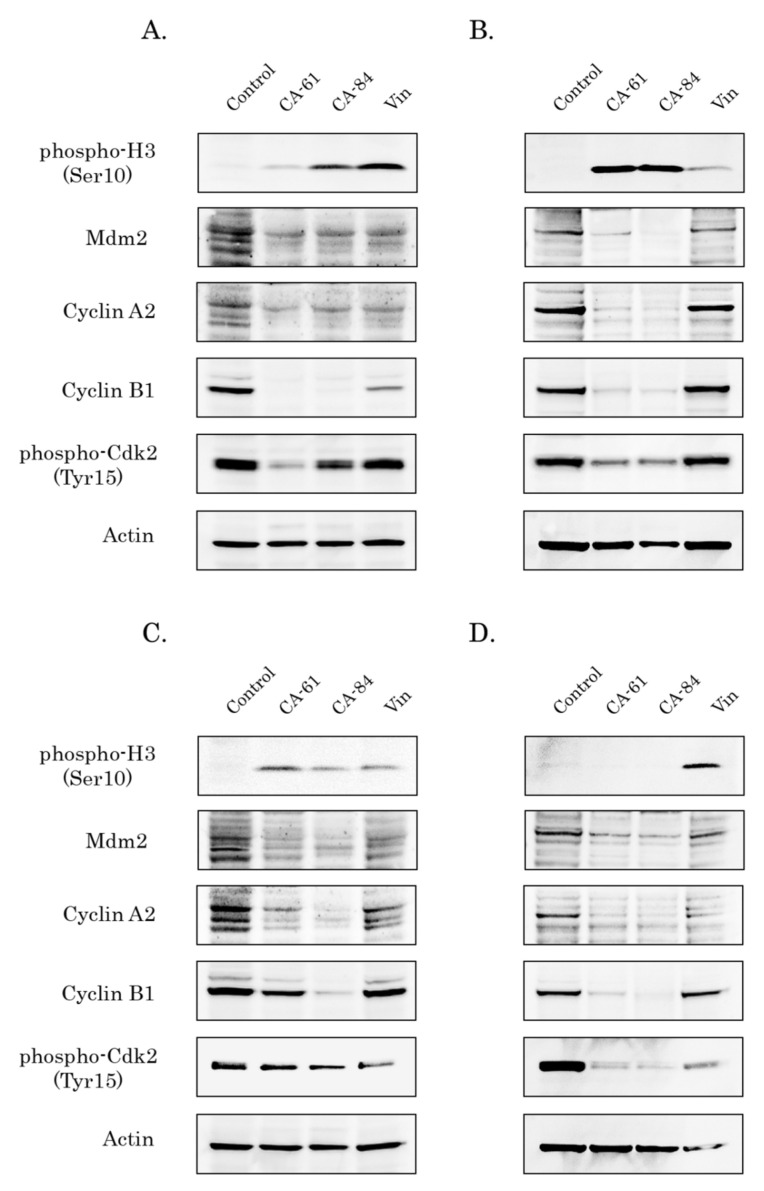
CA-61 and -84 dysregulate the expression of cell regulatory proteins in cancer cell lines. Immunoblot analysis for cyclins A2 and B1, Mdm2, phospho-Cdk2 (Tyr15), and phospho-H3 (Ser10) in HCC1806 breast cancer (**A**), MDA-MB-231 breast cancer (**B**), H1299 lung cancer (**C**), and PC-3 prostate cancer (**D**) cells after treatment with DMSO (negative control), CA-61, CA-84 (10 μM), and Vinblastine (Vin) (0.01 μM) for 48 h. Actin stain is used as a loading control.

In addition, Western blotting analysis also revealed a significant increase in pH3 Ser10 expression in the HCC1806 cancer cell lines treated with CA-61 and -84 (Figure 11A). Conversely, the expression of phospho-Cdk2 Tyr15, cyclin A2, and Mdm2 decreased after CA treatment. Similar changes in the cell-cycle-related proteins were observed for MDA-MB-231, and H1299 cells treated with CAs (Figure 11B,C, respectively). Of note, the changes in the expression of cell cycle regulatory proteins in Vin-treated cancer cells were much less prominent when compared to CA-treated cells, thereby revealing the highest potency of synthesized compounds to deregulate the progression of the cell cycle.

### 2.10. CAs Induce Apoptosis of Breast, Lung, and Prostate Cancer Cells

**Figure 12:**
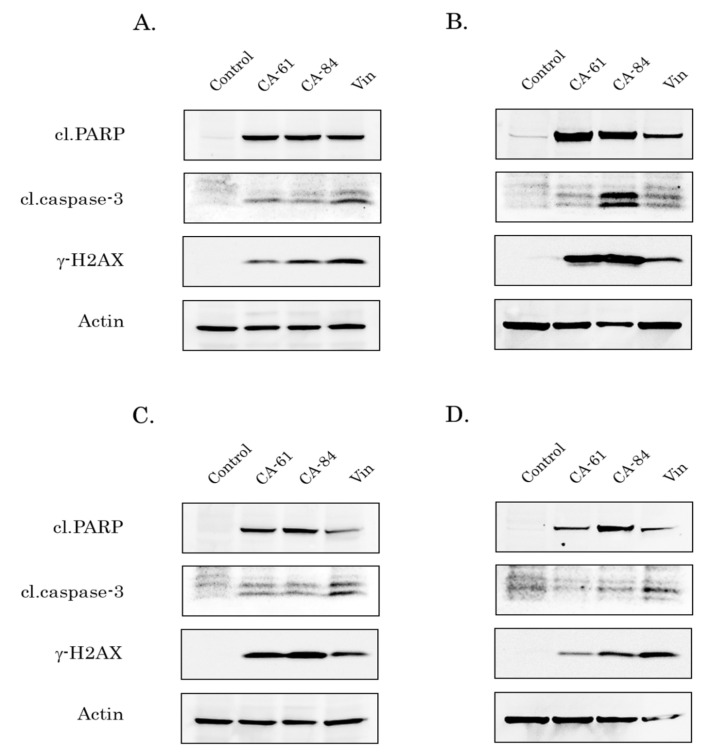
CA-61 and -84 induce apoptosis of epithelial cancer cell lines. Immunoblot analysis for apoptosis markers (cleaved forms of PARP and caspase-3), and γ-H2AX in HCC1806 breast cancer (**A**), MDA-MB-231 breast cancer (**B**), H1299 lung cancer (**C**), and PC-3 prostate cancer (**D**) cells after treatment with DMSO (negative control), CA-61, -84 (10 μM), and Vinblastine (Vin) (0.01 μM) for 48 h. Actin stain is used as a loading control.

To examine whether cytotoxic activities of CAs in vitro were due to the activation of the apoptosis of epithelial cancer cell lines as an outcome of their mitotic arrest, we assessed the expression of apoptotic markers (cleaved forms of caspase-3 and PARP) by Western blotting. Indeed, we found a significant increase in the expression of apoptotic markers in breast, lung, and prostate cancer cell lines after CA treatment in vitro. Of note, these compounds exhibited the highest pro-apoptotic activities for the majority of epithelial cancer cell lines, when compared with the tubulin-depolymerizing agent vinblastine (Vin). Densitometry analysis revealed the significant changes in the expression of apoptotic markers after the CA treatment of breast and lung cancer cell lines indicated above (Supplementary Figure S1). Again (consistent with molecular docking data), CA-84 exhibited the most potent pro-apoptotic activities against all cancer cell lines used in the present study (Figure 12). Of note, we also observed a substantial increase in the expression of H2AX phosphorylated at residue 139 (γ-H2AX) in CA-treated cells (Figure 11), thereby illustrating the potential DNA-damaging activities for these compounds. Further studies are needed to elucidate these activities of CAs. Of note, the pro-apoptotic effects of CAs were also similar to PTX, as shown in Supplementary Figure S2.

Collectively, the pro-apoptotic activities of CA-84 and -61 in vitro shown here were consistent with the tubulin polymerization data shown in Figure 5.

### 2.11. Anti-Tumor Activities of CAs in HCC1806 Xenografts

**Figure 13:**
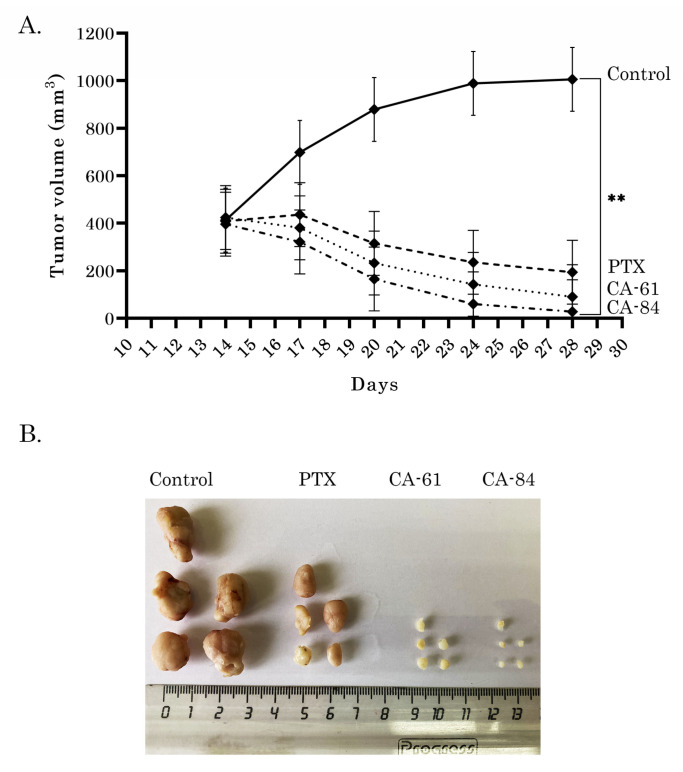
Antitumor effects of CAs and PTX in a nude mice xenograft tumor model. After HCC1806 breast cancer xenografts reached ~400 mm^3^ of volume (day 14), nude mice were randomized into 4 groups (n = 5) and administered i.p. 150 μL of vehicle (negative control), CA-61, CA-84 (2 mg/kg), or PTX (10 mg/kg). The animals were treated according to the schedule specified in Materials and Methods (**A**) The changes in the tumor sizes were calculated as a percentage of baseline. The tumor volume in each group was assessed by calipers and calculated as the length × width × width × 0.5. Results were expressed as the mean volume of tumors (mean ± SD, n = 4; **: *p* < 0.001, compared to control (vehicle-treated) animals. (**B**) Representative images of the final tumor volumes in each experimental group.

Lastly, we tested CAs (61 and 84) for their anti-tumor activities by using the HCC1806 breast cancer xenograft model. Xenograft tumors were allowed to reach ~400 mm^3^ volumes before the randomization of mice (n = 5) into treatment groups (day 14 after inoculation). PTX and CAs were diluted, as shown in the section *Chemical compounds*, and administered intraperitoneally every second day 5 times (days 14, 16, 18, 20, and 22). The doses of CAs and PTX used for this experiment were 2 and 10 mg/kg, respectively. As shown in Figure 13A, vehicle-treated mice (control) demonstrated a continuous increase in tumor size from the baseline. As expected, PTX inhibited the growth of breast cancer xenografts in a time-dependent manner. Strikingly, a substantial decrease in tumor size was observed in mice treated with CA-61 (Figure 13A). Similarly, a significant reduction in the size of HCC1806 xenografts was observed in CA-84-treated mice. Of note, we observed a statistical difference between the tumor volumes of PTX- and CA-84-treated mice (Figure 13A,B), illustrating that CA-84 had the higher antitumor activities when compared to the standard chemotherapeutic agent PTX. Importantly, no toxicity was observed in all animal groups over the whole period of the experiment (30 days). The representative images of the final tumor volumes from each experimental group are shown in Figure 12B. The H-E staining of tumor xenografts revealed the high antitumor potencies of both CAs, which were evidenced by the significant decrease in the number of cancer cells (Supplementary Figure S5).

## 3. Discussion

Despite the long-term and successful history of the clinical use of microtubule-targeting agents (MTAs) (mainly belonging to the taxanes and vinca alkaloids) for the therapy of a broad spectrum of human malignancies, MTA-based therapies have several serious limitations, including poor bioavailability and serious adverse effects (e.g., systemic and neural toxicity, myeloid toxicity, neutropenia, etc.). In addition, the limited benefits of MTAs-based therapies are also due to the rapid development of resistance to MTAs arising in tumors shortly after the initiation of chemotherapy as a part of their adaptive mechanisms, including an increased efflux of MTAs due to the upregulation of the P-glycoprotein [37], mutations of *TUB*, a selection of tubulin isotypes and its post-translational modifications [23,38], etc. This remains a major driving force to develop novel and potent MTAs effective against a broad spectrum of human malignancies.

It is known that pyrrole- and pyrazole-based derivatives are widely used for drug design to find out the potent candidates for anti-cancer agents. Multiple studies, including our own, report about their promising anti-proliferative and cytotoxic effects against various cancer cell lines, both in vitro and in vivo, due to their different modes of action. For example, a chemical compound containing pyrazole-3-carboxamide skeleton (N-(2-Chloropyridin-4-yl)-5-(4-methylphenyl)-1-(quinolin-2-yl)-1H-pyrazol-3-carboxamide), exhibited potent anti-proliferative and cytotoxic activities in vitro against different cancer cell lines, including human hepatocellular carcinoma, breast, and colon cancer and induced a robust cell cycle arrest in the G1/S-phase due to non-examined molecular mechanism [39]. 4,5-dihydro-1H-thieno [2′,3′:2,3]thiepino[4,5-c]pyrazole-3-carboxamide derivatives were effective against EGFR highly expressing human lung cancer A549 cell line in vitro and the molecular docking procedure revealed the similar EGFR-binding sites and modes of conformation (e.g., MET769) between the most effective derivative and gefitinib, a well-known EGFR inhibitor [40]. The derivatives of 4-benzoylamino-1H-pyrazole-3-carboxamide were recently shown as a potent CDK2 inhibitor that exhibited the highest anti-proliferative activities against cancer cell lines (e.g., A2058 melanoma and MV4-11 leukemia cell line) when compared to non-transformed human normal cell lines [41]. The derivatives of 1-Ethylpyrazole-3-carboxamide were reported as effective hypoxia-inducible factor (HIF)-1 inhibitors, suppressing the HIF-1-mediated hypoxia response, including carbonic anhydrase IX (CA IX) gene expression [42]. The ability of pyrazoles or pyrazoline derivatives to inhibit the activities of carbonic anhydrase isoenzymes (CAis) was extensively studied by the scientific group of Yamali C. and Gul H. In particular, they found that selective CAi IX or CAi XII enzymes’ inhibitory potencies and the cytotoxic activities of pyrazoles were highest for the pyrazoles containing benzenesulfonamide [43,44,45,46].

In contrast to the pyrazole-based compounds, multiple reports, including our own, illustrated the anti-cancer activities of pyrrole-containing agents due to their ability to interfere with tubulin in a dynamic state and the induction of apoptotic death as an outcome of the cell cycle arrest in the M-phase and mitotic catastrophe. One of the first reports illustrating a high potency of pyrrole-based compounds to destabilize the microtubules was published in 2007 and illustrated an activity of tetra-substituted brominated pyrrole, JG03-14, to promote the loss of cellular microtubules, the formation of aberrant mitotic spindles, the accumulation of cancer cells in the G2/M phase of the cell cycle, and the induction of mitochondria-mediated apoptotic pathways [47]. JG03-14 is an analogue of combretastatin A-4, a natural product obtained from the South Africa tree, *Combretum caffrum*, and is a well-known and prominent microtubule-destabilizing agent. However, the major obstacle for the clinical use of combretastatin A-4 is its cis-isoform of the olefinic bridge which is rapidly transformed to a less active trans-isoform. To prevent the transition from the cis- to the trans-isoform and stabilize the structure, the olefinic bridge of CA-4 was replaced by a pyrrole ring. This work was further extended and resulted in the generation of the JG03-14 analogs with potent anti-cancer activities [48]. Romagnoli R et al. recently found that cis-restricted analogs of combretastatin A-4 (CA-4) that contained a pyrrole nucleus interposed between the two aryl rings, also exhibited potent anti-proliferative activities against several cancer cell lines due to their ability to inhibit tubulin polymerization, block the cell cycle in the metaphase and activate mitochondria-mediated apoptosis pathways [49]. Of note, all reports shown above highlighted that the tubulin-depolymerization activity of the pyrrole-based compounds was due to their binding to the colchicine-binding site of the tubulin. Our scientific group also extensively studied the anti-cancer activities of the pyrrole-based 3-carboxylates by using both in vitro and in vivo models. We found for the first time that the cytotoxic and anti-proliferative activities of these compounds were caused by their abilities to interfere with the microtubule dynamic state by inhibiting the tubulin polymerization [25,26,27]. This in turn led to the robust cell cycle arrest of cancer cells in the M-phase and induced their apoptosis as an outcome of mitotic catastrophe. These findings encouraged our group to design novel pyrazole-3-carboxamides to explore potent tubulin polymerization inhibitors as attractive anti-cancer agents. Therefore, we synthesized 38 novel compounds and evaluated their potential ligand–protein interactions by molecular docking to find out which ligands exhibited the strongest binding effect to the tubulin. Based on the MTS-based data and computational analysis we found that two compounds (CA-61 and -84) were the most effective against all cancer cell lines in vitro, and exhibited the highest affinity to the colchicine-binding site of tubulin (Figure 1, Table 1, Supplementary Table S1). The results of the structure–activity relationship (SAR) demonstrated that the highest cytotoxic activities of CA-61 and -84 were due to the presence of amino group (shown as R1 in Supplementary Table S2), which was known to increase the binding affinity of the ligand and thereby enhanced its potency as a drug molecule. The presence of 4-(phenylamino)phenyl group in CA-84 led to an increase in the affinity of the ligand–receptor complex by forming hydrophobic interactions with Val238β, Cys241β, Leu242β,Leu248β, Leu252β, and Leu255β. The 4-ethylphenyl group in CA-166 was responsible for the most effective interactions with the hydrophobic amino acids (Val238β, Cys241β, Leu242β, Leu248β, Leu252β, and Leu255β) among the rest of aromatic functional groups. The oxygen atoms of 2-methyl-5-nitrophenyl formed H bonds with polar amino acids, LYS B:254 and ASN A:101. In CA-1489I the amino group in the R1 position promoted the formation of hydrogen bonds with GLN A:11, whereas the presence of the electron-withdrawing group Cl was attributed to an increase in the biological activity of this compound.

Next, we evaluated the ability of CA-61 and -84 to interfere with tubulin polymerization. Indeed, the data shown in Figure 5 revealed CAs as potent tubulin-targeting agents, inducing tubulin depolymerization in a dose-dependent manner. The abilities of CA-61 and -84 to disrupt the microtubules network were also confirmed by immunofluorescence staining, illustrating the diffuse gauze staining of α-tubulin in CA-treated cells, which was similar to cancer cells treated with Vin (Figure 7 and Figure 8). As an outcome of the microtubules network disruption after CA treatment, cancer cells exhibited the profound changes of cell cycle division and selectively accumulated in the M-phase, which was evidenced by the significant increase in the number of phospho-H3-Ser10-positive cells, as shown in Figure 10. This was consistent with changes in the morphology of CA-treated cells, illustrating the accumulation of the round-shaped cells (Figure 5) after CA treatment, and the flow cytometry data, which revealed a substantial increase in the number of cells in the G2/M phase (Figure 9). The abnormalities in the regulation of cell cycle progression after CA treatment were also evidenced by the changes in the expression of cell cycle regulatory proteins. Indeed, the Western blotting data shown in Figure 11, revealed an increase in phospho-H3-Ser10 expression in the breast and lung cancer cell lines treated with CAs, whereas the expression of Mdm2, phospho-Cdk-Tyr15, and both cyclins B1 and A2 substantially decreased after CA treatment (Figure 12). As a potential outcome of cell cycle arrest after CA treatment, epithelial cancer cell lines used in present study underwent apoptosis, which was evidenced by the increased expression of the cleaved forms of PARP and caspase-3, as shown in Figure 11. The high anti-cancer potency of CAs in vivo was demonstrated by using the HCC1806-based xenograft model, illustrating the significant tumor growth-inhibitory properties of both compounds tested in the present study (Figure 13).

Collectively, we showed that two novel pyrrole-based 3-carboxylates (CA-61 and -84) exhibited a high potency against epithelial cancer cells both in vitro and in vivo. The anti-cancer activities of these compounds were due to their tubulin-depolymerizing properties, which led to the disruption of the microtubules network in cancer cells, inducing a robust cell cycle arrest in the M-phase, and triggering apoptotic cell death as an outcome of mitotic catastrophe. This in turn illustrates that pyrrole-based 3-carboxylates targeting the colchicine-binding site in the tubulin might be considered as a prospective class of anti-cancer agents, and can therefore be used as a scaffold for the development of the novel class of MTAs. Further studies, including the assessment of the plasma stability of CAs, their gastrointestinal absorption, and blood–brain barrier permeability will provide important information about the perspective use of CAs for anti-cancer therapy. Another goal that must be met in the future is achieving the water solubility of CAs, which is an important property in drug development. Of note, a novel method for the synthesis of water-soluble α-aminopyrroles was developed recently [50] and might be helpful to improve the physical characteristics of pyrrole-based compounds exhibiting anti-cancer activities.

## 4. Materials and Methods

### 4.1. Chemical Compounds

Paclitaxel (PTX) and vinblastine (Vin) were purchased from Sigma, St Louis, Missouri, USA, and dissolved in 100% dimethyl sulfoxide (DMSO) (Sigma Aldrich, St. Louis, MO, USA). Thirty-eight novel CAs as potential microtubule destabilizing agents were synthesized in our laboratories according to the standard protocols, as shown in Scheme 1 and in previous studies [36]. For the in vitro studies, the compounds were dissolved in DMSO. For controls, the cells were treated with the same concentrations of solvent (0.1% final concentration (*v*/*v*). For in vivo experiments, a 40 mg/mL stock solution of PTX was prepared in DMSO and diluted before administration in a formulation, which contained a final concentration of 10% DMSO, 12.5% Cremophor EL, 12.5% ethanol, and 65% saline-based diluent (0.9% sodium chloride, 5% polyethylene glycol, 0.5% Tween 80), as shown in previous studies [51]. Similar to PTX, stock solutions of CAs were prepared in DMSO and diluted within the same formulation as shown above, thereby providing the identical control (i.e., vehicle-treated) group for CA- and PTX-treated animals.

### 4.2. Cell Lines and Culture Conditions

The following human tumor cell lines were used in the present study: HCC1806 and parental MDA-MB-231 breast cancer cell lines, PC-3 prostate cancer cell line, H1299 non-small cell lung cancer (NSCLC) cell line, and normal human diploid fibroblast cells (BJ). Cancer cell lines indicated above were obtained from the American Type Culture Collection (ATCC, Manassas, VA, USA). BJ tert human fibroblasts were kindly provided by O. Gjoerup, University of Pittsburgh, USA. All cell lines were maintained in Dulbecco’s modified Eagle’s medium or RPMI-1640 medium (PanEco, Moscow, Russia), supplemented with 10–15% fetal bovine serum (HyClone, Logan, UT, USA), 1% L-glutamine, 50 U/mL penicillin, and 50 μg/mL streptomycin (PanEco, Moscow, Russia), and cultured in a humidified atmosphere of 5% CO_2_ at 37 °C (LamSystems, Mass, Russia).

### 4.3. Antibodies

Primary antibodies raised against the following proteins were used for Western blotting: Mdm2, Cell Cycle and Apoptosis WB Cocktail (pCdk2(Tyr15)/pHH3(Ser10)/Actin/Cleaved PARP) (Abcam, Cambridge, MA, USA), pH2AX S139, cyclin B1 (Santa Cruz Biotechnology, Santa Cruz, CA, USA), and Cyclin A2, cleaved form of caspase-3 (Cell Signaling, Danvers, MA, USA). HRP-conjugated secondary antibodies for Western blotting were purchased from Santa Cruz Biotechnology. For immunofluorescence staining, we used primary antibodies: Phospho-Histone H3 (Ser10) conjugated with secondary antibodies, Alexa Fluor 488 (Cell Signaling, Danvers, Massachusetts, MA, USA) and α-tubulin (Sigma-Aldrich, St. Louis, MO, USA). Texas Red-conjugated secondary antibodies were used for immunofluorescence staining (Invitrogen, Carlsbad, CA, USA).

### 4.4. Cellular Survival MTS-Based Assay

Non-transformed BJ fibroblasts and cancer cells were plated into 96-well flat-bottomed plates (Corning Inc., Corning, NY, USA) and allowed to attach and grow for 24 h. The cells were then cultured for 48 h with indicated concentrations of CA-61 and -84, MTAs (positive control), or a solvent (DMSO). After 48 h of post-treatment, MTS reagent (Promega, Madison, WI, USA) was introduced into the cell culture medium for 1 h to assess the number of live cells. Cellular viability was analyzed at 492 nm on a MultiScan FC plate reader (Thermo Fisher Scientific, Waltham, MA, USA). Half-inhibitory concentration (IC_50_) was determined by using Excel software. All the data were normalized for the DMSO-treated (e.g., control) cells.

### 4.5. Tubulin Polymerization Assay

The impact of the CAs on the dynamic state of the tubulin polymerization was assessed by using the Tubulin Polymerization kit (Cytoskeleton Inc., Denver, CO, USA) as specified by the manufacturer. Results were obtained on a SpectraFluor Plus microplate reader (Tecan GmbH, Austria) and readings were taken every minute for 1 h (61 measurements in total).

### 4.6. Western Blotting

For the preparation of whole-cell extracts, the cells were lysed by RIPA buffer (25 mM Tris-HCl pH 7.6, 150 mM NaCl, 5 mM EDTA, 1% NP-40, 1% sodium deoxycholate, 0.1% SDS), and supplemented with the protease and phosphatase inhibitors. The cellular lysates were incubated in a buffer for 20 min at 4 °C and then clarified by centrifugation for 30 min at 13,000 rpm at 4 °C. Protein concentrations were measured by BCA assay (Thermo Fisher Scientific, Rockford, IL, USA). The samples containing 30 μg of protein were resolved on 4 to 12% Bis-Tris NuPAGE gel (Invitrogen, Carlsbad, CA, USA). SDS-PAGE was carried out at 4 °C for approximately 3 h using a constant voltage (80V) in 1X NuPAGE MOPS SDS running buffer (Invitrogen, Carlsbad, CA, USA). Protein transfer to a nitrocellulose membrane was performed by using 1X transfer buffer (25 mM Tris, 192 mM Glycine, 20% (*v*/*v*) methanol, (pH 8.3)) at 350 mA for 1.5 h at 4 °C. To block the nonspecific interactions, 5% non-fat dry milk was used in the antibody-dilution buffers. The nitrocellulose membrane was incubated with primary antibodies overnight at 4 °C, washed 3 times with 1xPBS, and further with HRP-conjugated secondary antibody for 1 h. Protein expression was detected by the chemiluminescence imaging system, Fusion Solo S (Vilber Lourmat, Collégien, France). Densitometry analysis of Western blotting images was performed by using the NIH Image J software (Bethesda, MD, USA).

### 4.7. Immunofluorescence Staining

To count the number of mitotic cells after CA treatment, cells were seeded on glass coverslips pre-coated with poly-L-lysine (Sigma-Aldrich, St. Louis, MO, USA) and allowed to attach for 48 h before treatment. After treatment (9 h incubation with drugs) the slides were gently washed with PBS (twice) and fixed with 4% formaldehyde diluted in 1X PBS for 15 min at room temperature. The cells were further washed three times with PBS (for 5 min each in the dark) and incubated in a blocking solution (1X PBS, 5% normal goat serum, 0.3% Triton X-100) for 60 min at room temperature. Then, the primary rabbit antibodies, Phospho-Histone H3 (Ser10) conjugated with Alexa Fluor 488 (Cell Signaling, Danvers, Massachusetts, USA) and diluted in Antibody Dilution Buffer (1X PBS, 1% bovine serum albumin, 0.3% Triton X-100), were added to the cells (overnight at 4 °C). The next day, the coverslips were gently washed three times with 1xPBS for 5 min each (in the dark) and stained with DAPI (Sigma-Aldrich, St. Louis, MO, USA) to visualize the nuclei. Further, the coverslips were mounted on glass slides and the cells were visualized on fluorescence microscope “Olympus BX63” (Olympus, Tokyo, Japan). Finally, the images were captured by using a Spot advanced imaging system.

To calculate the mitotic index, 5 low-power fields per experimental condition were analyzed. The data was further analyzed by the Kruskal–Wallis test followed by Mann–Whitney–Wilcoxon test with the Benjamini-Hochberg adjustment in R software (R Foundation for Statistical Computing, Vienna, Austria; Available online: https://www.R-project.org/, accessed on 5 April 2021).

For microtubules depolymerization experiments, the cells were pretreated with cold shock (culture media at 4 °C was added to the cells and the cell plates were put on ice during the treatment for 1 h) and further cultured in the pre-warmed medium for 2 h to promote tubulin polymerization. After washing with ice-cold PBS, the cells were fixed with a mixture of methanol and acetone (1:1 ratio) at—20 °C for 20 min, blocked, and permeabilized with 1X PBS, supplemented with 5% normal goat serum and 0.3% Triton X-100 for 60 min at room temperature. The cells were stained with primary mouse anti-α-tubulin antibodies (Sigma-Aldrich, St. Louis, MO, USA) overnight at 4 °C. The next day the cells were washed with 1xPBS and stained with Texas Red-conjugated secondary antibodies (Invitrogen, Carlsbad, CA, USA) for 60 min. After brief DAPI staining, the coverslips were mounted on glass slides, and cells were visualized as shown above.

### 4.8. Flow Cytometry

The distribution of cell cycle phases in HCC1806 cells was analyzed by Guava Muse Cell Analyzer using Cell Cycle Kit (Part Number: MCH100106) according to the manufacturer’s protocol. Five replicates per experimental condition were analyzed. The data were analyzed by the Kruskal–Wallis test followed by Mann–Whitney–Wilcoxon test with the Benjamini-Hochberg adjustment in R software (R Foundation for Statistical Computing, Vienna, Austria; Available online: https://www.R-project.org/, accessed on 23 May 2021).

### 4.9. Molecular Docking

To identify the potential binding sites of CAs on the tubulin, the molecular docking procedure was performed by using Schrodinger molecular modeling software (Schrödinger, Inc, New York, NY, USA, 2021). The Glide Docking SP and XP mode were used to find the tubulin-CAs supramolecular complex with minimal energy scoring function. The tubulin structure (no. 4O2B) was downloaded from the Protein Data Bank (4O2B. Available online: https://www.rcsb.org/structure/4O2B, accessed on 18 August 2021). The protein structure was prepared before docking using the Protein Preparation Wizard module. Initial ligand structures were optimized using standard preparation procedures in the LigPrep module. To determine the possible interactions of the most active CA with the tubulin molecule, the “blind docking”, without assigning a specific binding site, was performed. Potential binding sites for ligand docking were identified using the Site Map module. After defining binding sites, a Grid Box for each of them was generated by using the Receptor grid module.

The molecular mechanics/generalized Born surface area (MM-GBSA) method was used as a post-docking validation tool [52]. The binding energy calculated by MM-GBSA illustrated a reasonably positive correlation between the predicted and the experimental binding affinity. Based on the XP docked complex, we calculated the binding free energy (∆Gbind) of each ligand, including the solvation energy (∆Gsolv), using the implicit water model.

### 4.10. Xenograft Studies Models

Subcutaneous human tumor xenografts were generated via s.c. inoculation in flank areas of 5 to 8-week-old female nu/nu mice with 100 μL of 5 × 10^6^ HCC1806 breast cancer cells/mL suspensions in Dulbecco’s phosphate-buffered saline. The animal experimental protocols were approved by the Committee for Ethics of Animal Experimentation and the experiments were conducted in accordance with the Guidelines for Animal Experiments in N.N. Blokhin National Medical Research Center of Oncology. All s.c. tumors were allowed to reach ~400 mm^3^ volumes before randomization of mice into 4 treatment groups (day 14). The stocks of PTX and CAs were prepared as described in the *Chemical compounds* section. Mice were administered intraperitoneally 5 times every second day either 150 μL of vehicle (negative control), PTX (10 mg/kg), CA-61, and -84 (2 mg/kg). The administration schedule occurred on the following days: 14, 16, 18, 20, and 22. Tumor volumes were measured by the caliper every 3 days for 30 days in total. The average tumor volumes of each experimental group were calculated, and tumor growth curves were drawn accordingly. Animal weight and general health of the mice were also recorded. After, the mice were sacrificed, and the tumors were isolated, photographed, weighed and subjected to histopathologic examination. Formalin-fixed, paraffin-embedded (FFPE) tissues were sectioned at 4 μM for hematoxylin and eosin (H&E) staining. The images of the stained samples were captured using an Olympus BX63 microscope (Olympus, Tokyo, Japan).

## Figures and Tables

**Table 1:** Docking score (SP and XP Modes), MM-GBSA ΔGbind prime energy, ligand strain energy, H–bond, and hydrophobic interactions of the top-ranking CAs.

Compound	Glide Score XP (kcal/mol)	Glide Score XP (kcal/mol)	MM-GBSA ΔG_bind_ (kcal/mol)	Ligand Strain Energy	H-Bonds Interactions D(A,B)-A[X]	Hydrophobic Interaction (A,B)
CA-84	−9.903	−7.703	−16.82	11.22	OD1 (ASN A:101)-H13 [2.05] HZ3 (LYS B:254)-O3 [2.38] HZ3 (LYS B:254)-N4 [2.40]	VAL B:238 LEU B:248 CYS B:241 LEU B:242 MET B:259 ALA B:250 ALA B:354 ALA A:180 VAL A:181
CA-61	−9.390	−7.420	−16.60	12.95	HZ3 (LYS B:254)-O2 [2.53] HD22 (ASN A:101)-O2 [2.05] O (THR A:179)-H12 [2.29]	LEU B:248 ALA B:250 CYS B:241 LEU B:242 LEU B:255 ALA B:354 ALA B:316 ILE B:318
CA-166	−8.334	−7.009	−14.79	15.5	HD22 (ASN A:101)-O31 [2.41] NZ (LYS B:254)-O31 [2. 59] H (THR B:353)-O12 [2.09]	VAL B:238 LEU B:248 CYS B:241 LEU B:242 MET B:259 ALA B:250 ALA B:354 ALA A:180 VAL A:181
CA-59	−7.365	−6.359	−13.31	18.9	NZ (LYS B:254)-O31 [2. 78] H (THR B:353) -O12 [2.34]	LEU B:248 LEU B:255 ALA B:354 ILE A: 171 ALA B:250 VAL A:177 ALA A:180 VAL A:181 TYR A:224
CA-1489I	−8.823	−7.961	−14.63	28.7	HZ3 (LYS B:254)-O1 [2.20] HE22 (GNL A:11)-O3 [2.24] O (GNL B:247)-H3 [2.60] Halogen bound H (THR B:353) -Br23 [2.20]	LEU B:248 ALA B:250 LEU B:255 ALA B:354 ILE A: 171 VAL A:177 ALA A:180 VAL A:181 TYR A:224
CA-1488I	−7.125	−6.895	−10.24	13.41	HZ3 (LYS B:254)-O1 [2.18] O (GNL B:247)-H3 [2.63] Halogen bound H (THR B:353) -Br23 [2.22]	ALA A:180 VAL A:181 ALA B:354 ILE B:318 CYS B:241 MET B:259 LEU B:255 ALA B:316 ALA B:317
CA-1348I	−6.448	−3.446	−6.20	31.4	OD1 (ASN A:101)-H31 [2.26] HZ3 (LYS B:254)-N11 [2.72]	CYS B:241 MET B:259 LEU B:255 ALA B:354 ILE A: 171 VAL A:177 ALA A:180 VAL A:181 TYR A:224
BAL27862	−12.434	−10.987	−32.60	12.4	O (TYR B:202)-H46 [1.96] O (VAL B:239)-H45 [2.51] O (THR A:179)-H36 [2.76] HZ2 (LYS B:352)-N9 [2.00]	LEU B:248 LEU B:255 CYS B:241 ALA B:354 TYR B:202 VAL B:238 VAL B:351 ILE B: 347 PRO B:348

Abbreviations: D, donor; A, acceptor; X, length of interaction in Å; (A, B), Alpha or Beta-chain.

**Table 2:** IC_50_ values for CA-61 and -84 in non-transformed human fibroblasts (BJ tert) and epithelial cancer cell lines.

Cell Line	CA-61 (µM)	CA-84 (µM)
BJ tert	4.0 ± 0.5	4.2 ± 0.4
HCC1806	8.4 ± 0.3	7.2 ± 0.6
MDA-MB-231	3.2 ± 0.3	4.8 ± 0.6
H1299	2.8 ± 0.3	3.1 ± 0.5
PC-3	7.0 ± 0.4	6.8 ± 0.3

**Table 3:** Quantitative analysis of the cell-cycle distribution in HCC1806 breast cancer cells as measured by the flow cytometry described in Figure 7. Normally, distributed data are presented as mean ± standard deviation, non-normally distributed data are shown as median (interquartile range); * *p* < 0.05, ** *p* < 0.01.

	G0/G1	S	G2/M
Control	37.04 ± 2.17	18.80 (18.5–18.9)	42.26 ± 3.03
CA-61	22.84 ± 2.35 **	23.38 ± 2.68 *	51.26 ± 3.65 *
CA-84	24.10 (24–24.5) **	23.14 ± 0.69 *	68.30 (67.6–69)*
PTX	1.90 ± 0.85 **	20.40 ± 2.47	68.24 ± 1.80 *
Vin	30.48 ± 1.54 **	19.56 ± 0.74	47.12 ± 1.34 *

## Data Availability

Not applicable.

## Supplementary Materials

The following are available online. Figure S1: Densitometry analysis of cleaved PARP and caspase-3 in cancer cell lines treated with CAs and Vin. bars, SD. * *p* < 0.05; ** *p* < 0.01. Figure S2: CA-61 and 84 induce apoptosis of epithelial cancer cell lines. Immunoblot analysis for apoptosis markers (cleaved forms of PARP and caspase-3) in HCC1806 breast cancer (A), MDA-MB-231 breast cancer (B), H1299 lung cancer (C), and PC-3 prostate cancer (D) cells after treatment with DMSO (negative control), CA-61, -84 (10 μM), and Paclitaxel (0.1 μM) for 48 h. Actin stain is used as a loading control. Figure S3: Densitometry analysis of cleaved PARP and caspase-3 in cancer cell lines (as shown in Supplementary Figure S2) treated with CAs and PTX. bars, SD. ** *p* < 0.05; *** *p* < 0.01. Supplementary Figure S4. Spectra characteristics of CAs. Supplementary Figure S5. Representative images of the HCC1806 xenografts treated with a solvent (control), paclitaxel (PTX), CA-61, and -84. Supplementary Scheme S1: Synthetic pathway of the derivatives of 2-aminopyrrole. Table S1: Characteristics of pyrrole-based compounds and IC50 values in MDA-MB-231 breast cancer cell line. Table S2: Structure-activity relationship (SAR)-based analysis of the features of the molecules in the dataset with observed activities.

## Sample Availability

Samples of the compounds are not available from the authors.
