# Supplementary material for: The Design, Synthesis, and Biological Activities of Pyrrole-Based Carboxamides: The Novel Tubulin Inhibitors Targeting the Colchicine-Binding Site
Source: Molecules. 2021 Sep 24;26(19):5780. doi: 10.3390/molecules26195780 (PMC8510300; doi:10.3390/molecules26195780)
Supplement: Supplementary file 1 [file molecules-26-05780-s001.zip › molecules-1347536-SM updated.pdf]

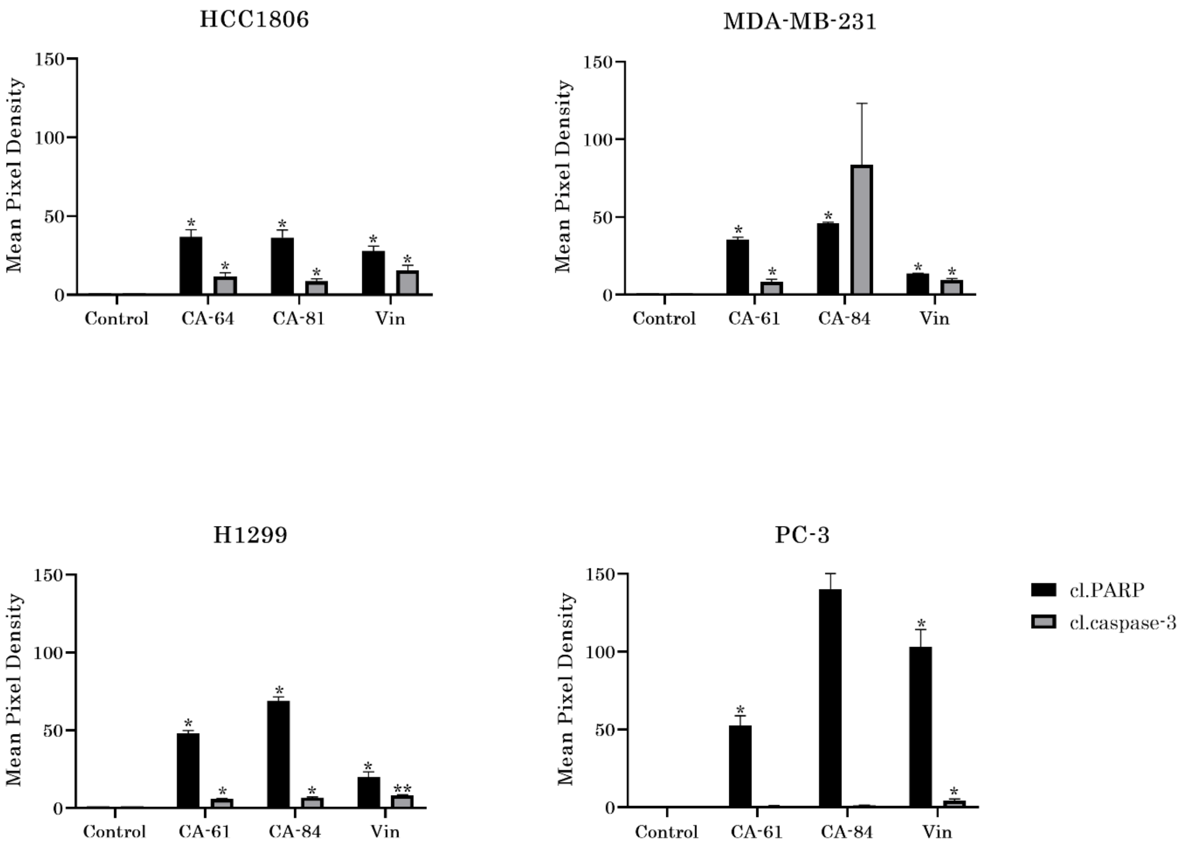

Supplementary Figure 1. Densitometry analysis of cleaved PARP and caspase-3 in cancer cell lines treated with CAs and Vin. bars, SD. * $p < 0.05$; ** $p < 0.01$.

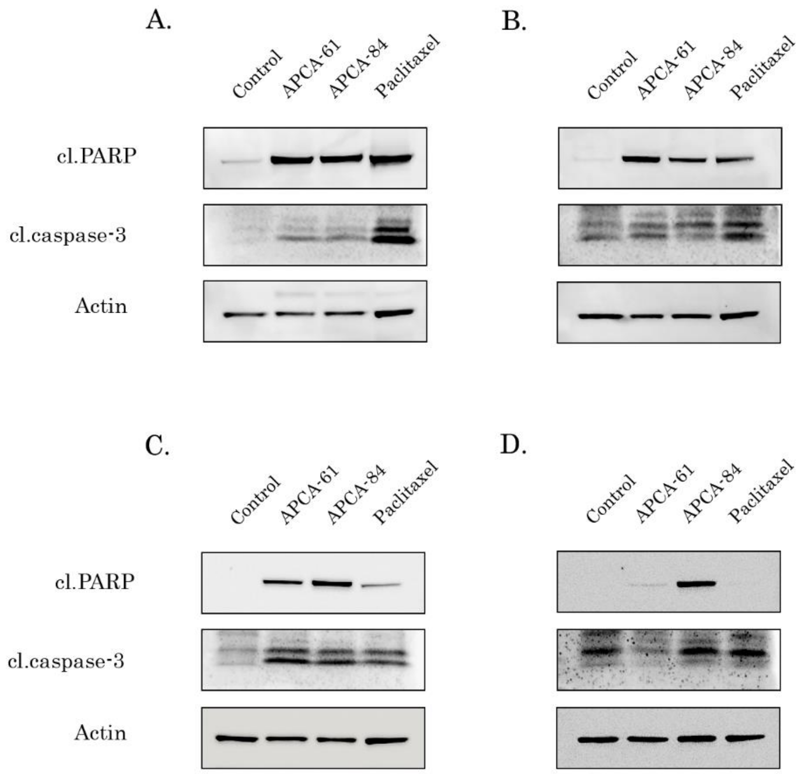

Supplementary Figure 2. CA-61 and 84 induce apoptosis of epithelial cancer cell lines. Immunoblot analysis for apoptosis markers (cleaved forms of PARP and caspase-3) in HCC1806 breast cancer (A), MDA-MB-231 breast cancer (B), H1299 lung cancer (C), and PC-3 prostate cancer (D) cells after treatment with DMSO (negative control), CA-61, -84 (10 μM), and Paclitaxel (0.1 μM) for 48 h. Actin stain is used as a loading control.

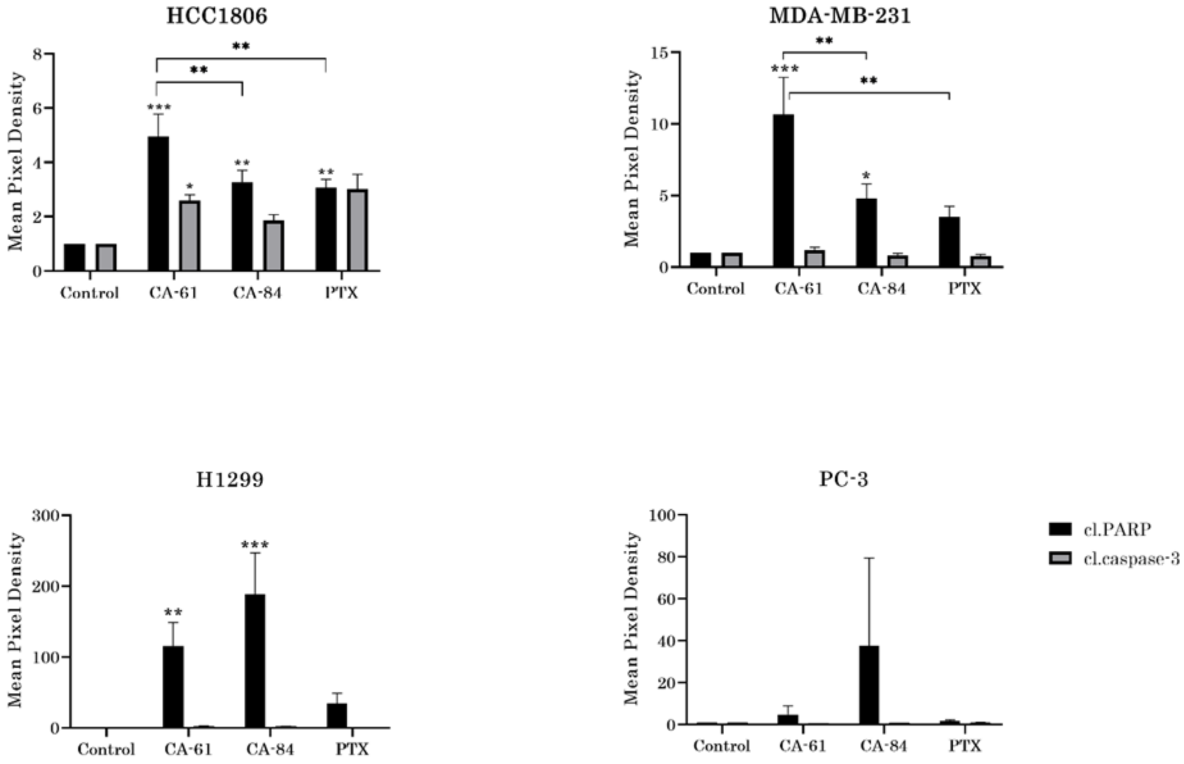

Supplementary Figure 3. Densitometry analysis of cleaved PARP and caspase-3 in cancer cell lines (as shown in Supplementary Figure 2) treated with CAs and PTX. bars, SD. ** $p < 0.05$; *** $p < 0.01$.

Ethyl 2-amino-1-(2-methyl-5-nitrophenyl)-4-oxo-5-(2-oxo-2-phenylethylidene)-4,5-dihydro-1H-pyrrole-3-carboxylate (CA-33).

IR spectre, ν, cm–1: 3304 (NH), 1697 (COOEt), 1648, 1600 (C=O,C=C). NMR 1H spectra (DMSO-d6), δ, ppm: 1.10 t (3H, CMe), 2.24 s (3H, Me), 3.99 q (2H, CH2), 5.40 s (1H, CH), 7.80 m (8H, Ar), 8.24 s (1H, NH), 8.32 s (1H, NH).

Ethyl-2-amino-1-(2-methyl-5-nitrophenyl)-4-oxo-5-(2-oxo-2-(p-tolyl)ethylidene)-4,5-dihydro-1H-pyrrole-3-carboxylate (CA-208).

IR spectre, ν, cm–1: 3303, 3168 (NH), 1672 (COOEt), 1646, 1608 (C=O, C=C). NMR 1H spectra (DMSO-d6), δ, ppm: 1.09 t (3H, C-Me), 2.25 s (3H, Me), 2.28 s (3H, Me), 3.92 q (2H, CH2), 5.34 s (1H, CH), 7.80 m (7H, Ar), 8.24 s (1H, NH), 8.34 s (1H, NH).

Ethyl-2-amino-5-(2-(4-chlorophenyl)-2-oxoethylidene)-1-(2-methyl-5-nitrophenyl)-4-oxo-4,5-dihydro-1H-pyrrole-3-carboxylate (CA-59).

IR spectre, ν, cm–1: 3296, 3172 (NH), 1670 (COOEt), 1650, 1596 (C=O, C=C). NMR 1H spectra (DMSO-d6), δ, ppm: 1.16 t (3H, C-Me), 2.29 s (3H, Me), 4.00 q (2H, CH2), 5.43 s (1H, CH), 7.80 m (7H, Ar), 8.34 s (1H, NH), 8.40 s (1H, NH).

Ethyl-2-amino-5-(2-(2,4-dimethylphenyl)-2-oxoethylidene)-1-(2-methyl-5-nitrophenyl)-4-oxo-4,5-dihydro-1H-pyrrole-3-carboxylate (CA-166).

IR spectre, ν, cm–1: 3296 (NH), 1676 (COOEt), 1650, 1612 (C=O, C=C). NMR 1H spectra (DMSO-d6), δ, ppm: 1.16 t (3H, CMe), 2.26 s (3H, Me), 2.26 s (3H, Me), 2.26 s (3H, Me), 4.0 q (2H, CH2), 5.43 s (1H, CH), 7.80 m (8H, Ar), 8.32 s (1H, NH), 8.40 s (1H, NH)

Ethyl-2-amino-1-(2-methyl-5-nitrophenyl)-5-(2-(naphthalen-1-yl)-2-oxoethylidene)-4-oxo-4,5-dihydro-1H-pyrrole-3-carboxylate (CA-90).

IR spectre, ν, cm–1: 3288, 3216 (NH), 1676 (COOEt), 1646, 1612 (C=O, C=C). NMR 1H spectra (DMSO-d6), δ, ppm: 1.10 t (3H, C-Me), 2.31 s (3H, Me), 4.02 q (2H, CH2), 5.71 s (1H, CH), 7.90 m (10H, Ar), 8.73 s (1H, NH) 8.80 s (1H, NH).

Ethyl-2-amino-1-(4-bromophenyl)-5-(3,3-dimethyl-2-oxobutyl)-4-oxo-5-(phenylthio)-4,5-dihydro-1H-pyrrole-3-carboxylate (CA-1474I)

NMR 1H spectra (DMSO-d6), δ, ppm: 0.80 s (9H, t-Bu), 1.17 t (3H, CH2CH3, J 7.0 Hz), 2.95 d (2H, CH2, J 18.6 Hz), 3.18 d (2H, CH2, J 18.6 Hz), 4.04 м (2H, CH2CH3), 7.46 m (9Har+1H, NH), 7.80 widened s (1H, NH).

Ethyl-2-amino-1-(4-bromophenyl)-5-((4-chlorophenyl)thio)-5-(3,3-dimethyl-2-oxobutyl)-4-oxo-4,5-dihydro-1H-pyrrole-3-carboxylate (CA-1488I)

NMR 1H spectra (DMSO-d6), δ, ppm: 0.80 s (9H, t-Bu), 1.17 t (3H, CH2CH3, J 7.0 Hz), 2.97 d (2H, CH2, J 18.6 Hz), 3.19 d (2H, CH2, J 18.6 Hz), 4.08 m (2H, CH2CH3), 7.48 m (8Har+1H, NH), 7.82 widened s (1H, NH).

2-amino-1-(4-bromophenyl)-5-((4-chlorophenyl)thio)-5-(3,3-dimethyl-2-oxobutyl)-4-oxo-4,5-dihydro-1H-pyrrole-3-carboxamide (CA-1489I)

NMR 1H spectra (DMSO-d6), δ, ppm: 0.80 s (9H, t-Bu), 3.04 d (2H, CH2, J 18.6 Hz), 3.22 d (2H, CH2, J 18.6 Hz), 6.62 widened s (1H, NH), 7.12 widened s (1H, NH), 7.48 м (8Har), 7.83 widened s (1H, NH), 8.23 widened s (1H, NH)

Ethyl-2-amino-1-(4-chlorophenyl)-5-(3,3-dimethyl-2-oxobutylidene)-4-oxo-4,5-dihydro-1H-pyrrole-3-carboxylate (CA-1453I)

NMR 1H spectra (DMSO-d6), δ, ppm: 1.06 s (9H, t-Bu), 1.21 t (3H, Me, J7.1Hz), 4.14 q (2H, CH2, J7.1Hz), 5.44 s (1H, CH), 7.45 d (2Har, J8.4Hz), 7.64 d (2Har, J8.4Hz), 8.09 widened s (1H, NH), 8.15 widened s (1H, NH).

Amino-1-benzamido-5-(3,3-dimethyl-2-oxobutylidene)-4-oxo-N-(p-tolyl)-4,5-dihydro-1H-pyrrole-3-carboxamide (CA-1265I)

NMR 1H spectra (DMSO-d6), δ, ppm: 0.98 s (9H, t-Bu), 2.28 s (3H, Me), 6.51 s (1H, CH), 7.14 d (2H, ar., J 8.1 Hz), 7.50 d (2H, ar., J8.1 Hz), 7.57 m (3H, ar.), 7.94 d (2H, ar., J 7.5 Hz), 8.77 widened s (1H, NH), 9.39 s (1H, NH), 9.99 s (1H, NH), 10.94 s (1H, NH).

Ethyl-2-amino-5-(3,3-dimethyl-2-oxobutylidene)-1-(2-hydroxybenzamido)-4-oxo-4,5-dihydro-1H-pyrrole-3-carboxylate (CA-1348I)

NMR 1H spectra (DMSO-d6), δ, ppm: 0.93 s (9H, t-Bu), 1.24 t (3H, Me, J7.1Hz), 4.18 q (2H, CH2, J7.1Hz), 6.33 s (1H, CH), 7.36 m (4H, ar.), 8.38 widened s (1H, NH), 9.05 s (1H, NH), 10.65 s (1H, OH), 11.35 s (1H, NH).

2-Amino-5-(3,3-dimethyl-2-oxobutylidene)-1-(2,2-diphenylvinyl)-4-oxo-4,5-dihydro-1H-pyrrole-3-carbonitrile (CA-23)

NMR 1H spectra (DMSO-d6), δ, ppm: 0.93 s (9H, t-Bu), 5.93 s (1H, CH), 7.51 m (7Har), 7.64 m (3Har), 8.73 widened s (1H, NH2), 9.09 widened s (1H, NH2).

CA-610I NMR ^{1}H spectra (DMSO-d6), δ, ppm: 0.91 s (t-Bu), 1.23 t (3H, CMe), 2.40 s (3H, Me), 4.21 q (2H, CH_2), 6.32 s (1H, CH), 7.32 d (2H, Ar), 7.83 d (2H, Ar), 8.44 s (1H, NH), 9.20 s (1H, NH), 10.86 s (1H, NH).

CA-1296I NMR ^{1}H spectra (DMSO-d6), δ, ppm: 0.83 s (9H, *t*-Bu), 1.23 t (3H, $CH_2\underline{CH_3}$, *J* 7.1 Hz), 2.74 d (2H, CH_2, *J* 18.7 Hz), 3.11 d (2H, CH_2, *J* 18.7 Hz), 4.14 q (2H, $\underline{CH_2}CH_3$, *J* 7.1 Hz), 7.52 m (8H_{ar},1H, NH), 7.83 widened s (1H, NH), 8.03 widened s (1H, NH), 9.60 d (1H, NH, *J* 5.0 Hz).

CA-1283I NMR ^{1}H spectra (DMSO-d6), δ, ppm: 0.82 s (9H, *t*-Bu), 1.25 t (3H, $CH_2\underline{CH_3}$, *J* 7.1 Hz), 2.62 d (2H, CH_2, *J* 18.7 Hz), 3.05 d (2H, CH_2, *J* 18.7 Hz), 3.16 s (3H, OCH_3), 4.15 q (2H, $\underline{CH_2}CH_3$, *J* 7.1 Hz), 7.06 d (2H_{ar}, *J* 8.6 Hz), 7.7 d (2H_{ar}, *J* 8.6 Hz), 8.17 widened s (1H, NH), 8.22 widened s (1H, NH).

CA -1282I NMR ^{1}H spectra (DMSO-d6), δ, ppm: 0.89 s (9H, *t*-Bu), 1.23 t (3H, $CH_2\underline{CH_3}$, *J* 7.0 Hz), 2.80 d (2H, CH_2, *J* 18.7 Hz), 3.27 d (2H, CH_2, *J* 18.7 Hz), 3.46 d (2H, CH_2, *J* 14.4 Hz), 3.62 d (2H, CH_2, *J* 14.4 Hz), 4.14 d (2H, $\underline{CH_2}CH_3$, *J* 7.0 Hz), 7.35 m (9H_{ar}), 7.94 widened s (2H, 2NH), 10.07 widened s (1H, NH)

CA -1288I NMR ^{1}H spectra (DMSO-d6), δ, ppm: 0.89 s (9H, *t*-Bu), 2.84 s (2H, CH_2, *J* 18.6 Hz), 3.28 d (2H, CH_2, *J* 18.6 Hz), 3.42 d (2H, CH_2, *J* 14.4 Hz), 3.58 d (2H, CH_2, *J* 14.4 Hz), 6.81 widened s (1H, NH), 7.38 m (9H_{ar}+1H, NH), 8.51 widened s (1H, NH), 7.88 widened s (1H, NH), 10.11 s (1H, NH)

CA -1519I NMR ^{1}H spectra (DMSO-d6), δ, ppm: 1.22 t (3H, $CH_2\underline{CH_3}$, *J* 7.0 Hz), 4.16 q (2H, $\underline{CH_2}CH_3$, *J* 7.0 Hz), 6.75 s (CH), 7.35 m (12H_{ar}), 8.38 s (1H, NH), 9.09 widened s (1H, NH), 10.93 s (1H, NH),

CA -581I NMR ^{1}H spectra (DMSO-d6), δ, ppm: 1.18 t (3H, $CH_2\underline{CH_3}$, *J* 7.1 Hz), 4.15 q (2H, $\underline{CH_2}CH_3$, *J* 7.1 Hz), 6.64 s (CH), 7.54 m (7H_{ar}), 8.48 s (1H, NH), 8.62 m (2H_{ar}), 9.20 s (1H, NH), 11.31 s (1H, NH).

CA -1222I NMR ^{1}H spectra (DMSO-d6), δ, ppm: 1.06 s (9H, *t*-Bu), 2.24 s (3H, Me), 5.54 s (1H, CH), 7.77 d (1H_{ar}, *J* 8.4 Hz), 8.18 widened s (1H, NH), 8.34 m (2H_{ar}), 8.96 widened s (1H, NH)

CA -26 NMR ^{1}H spectra (DMSO-d6), δ, ppm: 1.07 s (9H, *t*-Bu), 1.24 t (3H, $CH_2\underline{CH_3}$, *J* 7.0 Hz), 4.17 q (2H, $\underline{CH_2}CH_3$, *J* 7.0 Hz), 5.22 s (1H, CH), 7.70 m (4H_{ar}), 8.12 widened s (1H, NH), 8.26 s (1H, NH).

CA -46 NMR ^{1}H spectra (DMSO-d6), δ, ppm: 1.08 s (9H, *t*-Bu), 1.25 t (3H, Me, *J* 7.4 Hz), 4.05 q (2H, CH_2, *J* 7.4 Hz), 5.02 s (1H, CH), 7.23-8.12 m (6H, Ar+NH_2).

CA -20 NMR ^{1}H spectra (DMSO-d6), δ, ppm: 0.98 s (9H, *t*-Bu), 1.21 t (3H, Me, *J* 7.2 Hz), 4.03 q (2H, CH_2, *J* 7.2 Hz), 5.31 s (1H, CH), 7.49-7.85 m ($3H_{ar}$), 8.04 widened s (1H, NH), 8.26 widened s (1H, NH).

CA -28 NMR ^{1}H spectra (DMSO-d6), δ, ppm: 1.05 s (9H, *t*-Bu), 1.21 t (3H, Me, *J* 7.3 Hz), 4.09 q (2H, CH_2, *J* 7.3 Hz), 5.32 s (1H, CH), 7.42-8.28 m (5H, Ar+NH_2).

CA -631I NMR ^{1}H spectra (DMSO-d6), δ, ppm: 0.99 s (9H, *t*-Bu), 1.16 t (3H, Me, *J* 7.3 Hz), 4.05 q (2H, CH_2, *J* 7.3 Hz), 5.31 s (1H, CH), 7.57-8.2 m (5H, Ar+NH_2).

CA -959I NMR ^{1}H spectra (DMSO-d6), δ, ppm: 1.09 s (9H, *t*-Bu), 2.24 s (3H, Me), 5.69 s (1H, CH), 7.71 m ($5H_{ar}$), 8.46 s (1H, NH), 8.63 s (1H, NH), 11.21 s (1H, NH).

CA -915I NMR ^{1}H spectra (DMSO-d6), δ, ppm: 1.15 s (9H, *t*-Bu), 5.79 s (1H, CH), 7.36 m ($8H_{ar}$), 8.10 widened s (1H, NH), 9.05 s (1H, NH), 11.45 s (1H, NH).

CA -957I NMR ^{1}H spectra (DMSO-d6), δ, ppm: 0.91 s (9H, *t*-Bu), 5.47 s (1H, CH), 7.33 m ($6H_{ar}$), 8.45 widened s (1H, NH), 8.71 s (1H, NH), 11.35 widened s (1H, NH).

CA -958I NMR ^{1}H spectra (DMSO-d6), δ, ppm: 1.16 s (9H, *t*-Bu), 5.87 s (1H, CH), 7.23 m ($8H_{ar}$), 8.27 widened s (1H, NH), 11.24 s (1H, NH), 11.47 s (1H, NH).

CA -974I NMR ^{1}H spectra (DMSO-d6), δ, ppm: 1.07 s (9H, *t*-Bu), 5.72 s (1H, CH), 7.67 m ($5H_{ar}$), 8.53 s (1H, NH), 8.80 s (1H, NH), 11.33 widened s (1H, NH).

CA -1473I NMR ^{1}H spectra (DMSO-d6), δ, ppm: 1.61 t (3H, Me, *J* 7.1 Hz), 4.23 q (2H, CH_2, *J* 7.1 Hz), 6.70 s (1H, CH), 7.62 m ($7H_{ar}$), 8.47 m ($1H_{ar}$), 8.73 m ($1H_{ar}$), 8.90 widened s (1H, NH), 9.16 widened s (1H, NH), 11.20 s (1H, NH).

Supplementary Figure 4. Spectra characteristics of CAs.

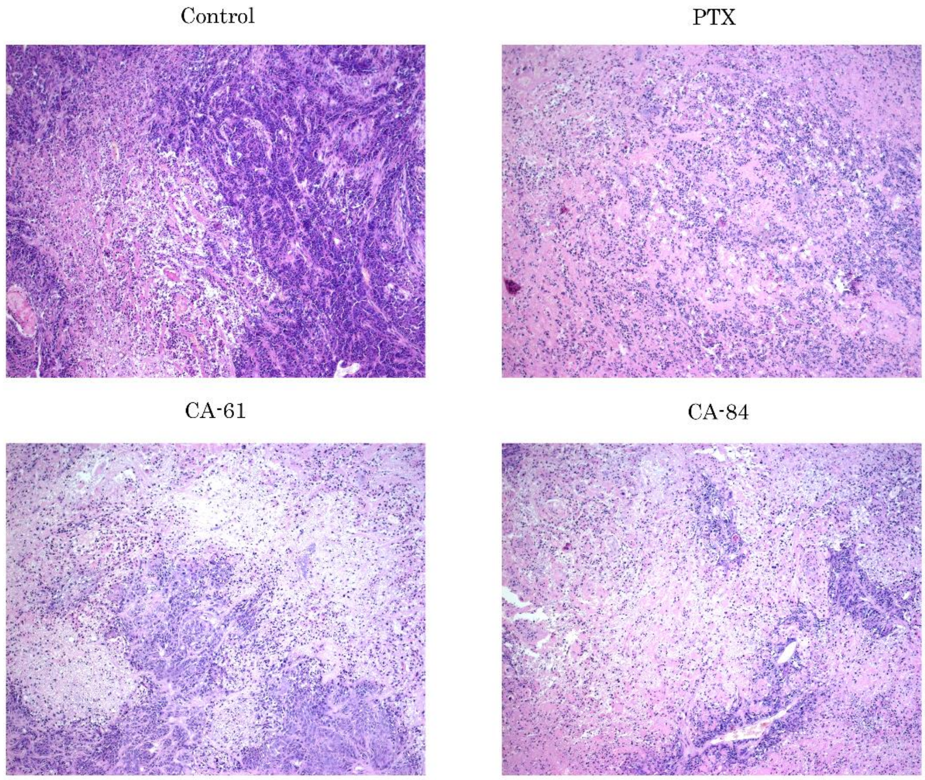

Supplementary Figure 5. Representative images of the HCC1806 xenografts treated with a solvent (control), paclitaxel (PTX), CA-61, and -84.

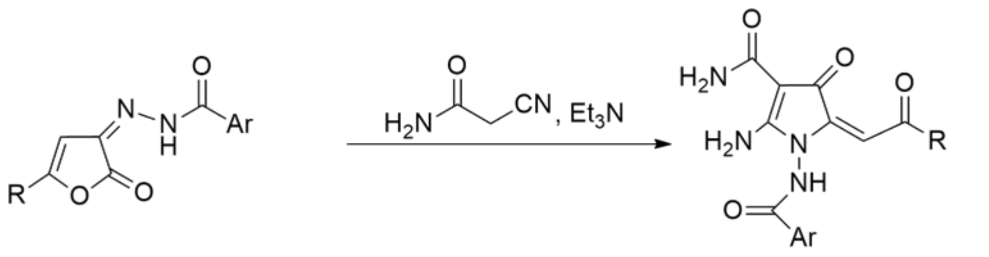

CA-61-a, CA-84-a **CA-61, CA-84**

Ar = C_6H_5 (CA-61-a, CA-61), 2-$C_6H_5NHC_6H_4$ (CA-84-a, CA-84);
R = naphthalen-2-yl (CA-61-a, CA-61), t-Bu (CA-84-a, CA-84)

Supplementary Scheme 1. Synthetic pathway of the derivatives of 2-aminopyrrole

Supplementary Table 1. Characteristics of pyrrole-based compounds and IC_{50} values in MDA-MB-231 breast cancer cell line

№	Structure and molecule weight	Brutto formula	Abbreviation	IC_{50} (μM)
1	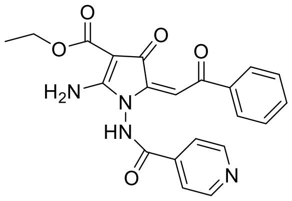<br>Molecular Weight: 406,40	$C_{21}H_{18}N_4O_5$	CA-581I	>100
2	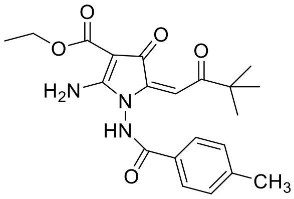<br>Molecular Weight: 399,45	$C_{21}H_{25}N_3O_5$	CA-610I	66 ± 5.2
3	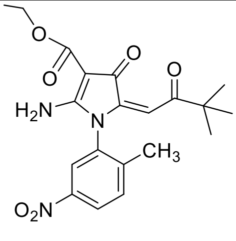<br>Molecular Weight: 401,42	$C_{20}H_{23}N_3O_6$	CA-631I	>100
4	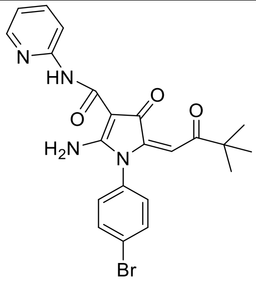<br>Molecular Weight: 469,34	$C_{22}H_{21}BrN_4O_3$	CA-915I	>100
5	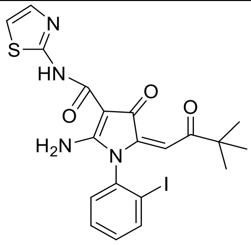<br>Molecular Weight: 522,36	$C_{20}H_{19}IN_4O_3S$	CA-957I	76.7 ± 3.9
6	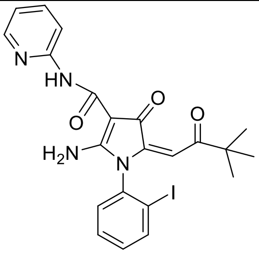<br>Molecular Weight: 516,34	$C_{22}H_{21}IN_4O_3$	CA-958I	>100

7	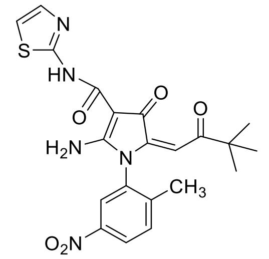 Molecular Weight: 455,49	$C_{21}H_{21}N_5O_5S$	CA-959I	>100
8	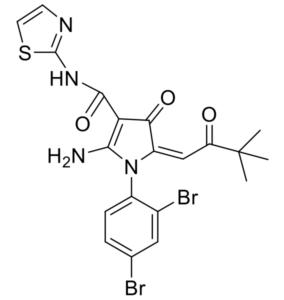 Molecular Weight: 554,26	$C_{20}H_{18}Br_2N_4O_3S$	CA-974I	82.9 ± 18.5
9	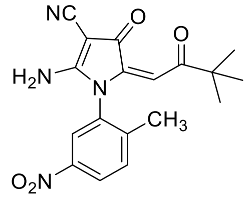 Molecular Weight: 354,37	$C_{18}H_{18}N_4O_4$	CA-1222I	>100
10	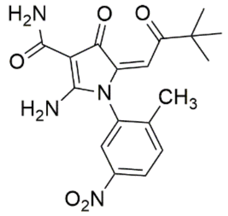 Molecular Weight: 372,38	$C_{18}H_{20}N_4O_5$	CA-1223I	>100
11	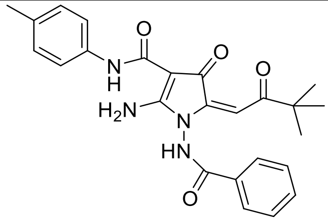 Molecular Weight: 446,51	$C_{25}H_{26}N_4O_4$	CA-1265I	40 ± 7.8
12	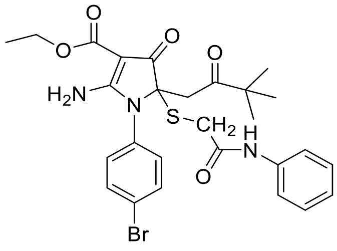 Molecular Weight: 588,52	$C_{27}H_{30}BrN_3O_5S$	CA-1282I	>100

13	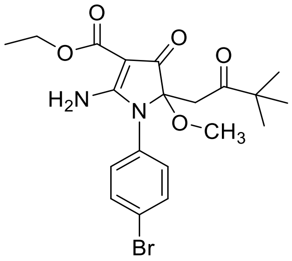 Molecular Weight: 453,33	$C_{20}H_{25}BrN_2O_5$	CA-1283I	>100
14	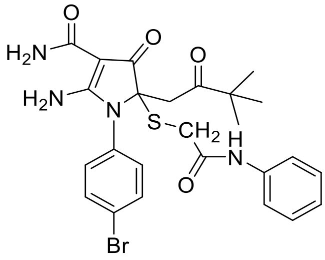 Molecular Weight: 559,48	$C_{25}H_{27}BrN_4O_4S$	CA-1288I	>100
15	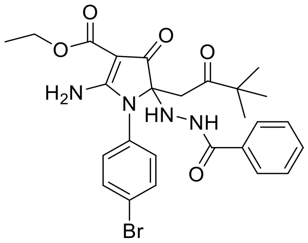 Molecular Weight: 557,45	$C_{26}H_{29}BrN_4O_5$	CA-1296I	>100
16	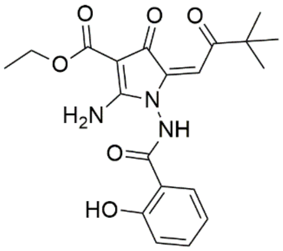 Molecular Weight: 401,42	$C_{20}H_{23}N_3O_6$	CA-1348I	21.3 ± 4
17	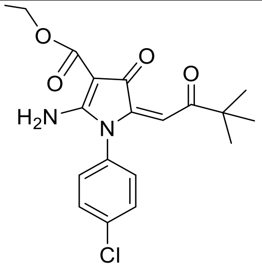 Molecular Weight: 376,84	$C_{19}H_{21}ClN_2O_4$	CA-1453I	58.7 ± 3.2
18	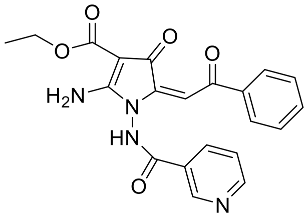 Molecular Weight: 406,40	$C_{21}H_{18}N_4O_5$	CA-1473I	87.3 ± 5.9

19	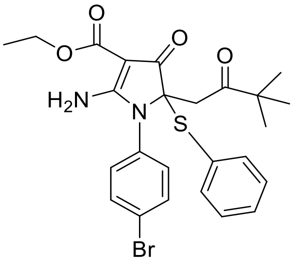<br>Molecular Weight: 531,47	$C_{25}H_{27}BrN_2O_4S$	CA-1474I	57.1 ± 6.6
20	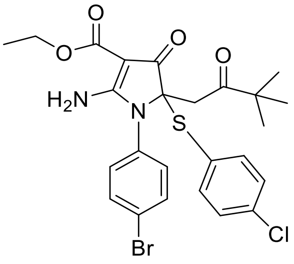<br>Molecular Weight: 565,91	$C_{25}H_{26}BrClN_2O_4S$	CA-1488I	18.5 ± 0.5
21	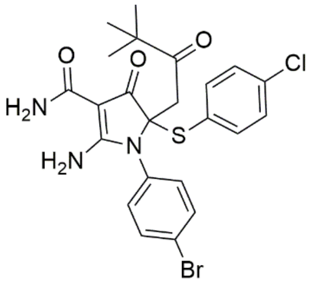<br>Molecular Weight: 536,87	$C_{23}H_{23}BrClN_3O_3S$	CA-1489I	13 ± 0.4
22	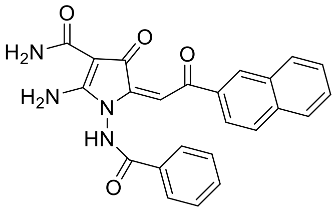<br>Molecular Weight: 426,43	$C_{24}H_{18}N_4O_4$	CA-61	3.2 ± 0.3
23	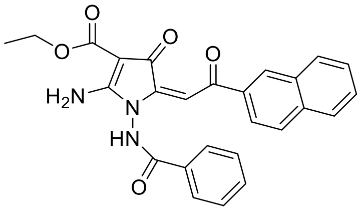<br>Molecular Weight: 455,47	$C_{26}H_{21}N_3O_5$	CA-1519I	>100
24	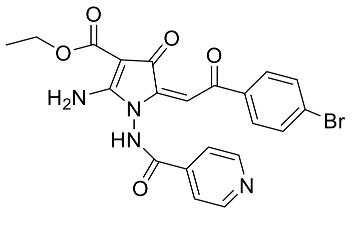<br>Molecular Weight: 485,29	$C_{21}H_{17}BrN_4O_5$	CA-1573I	>100

25	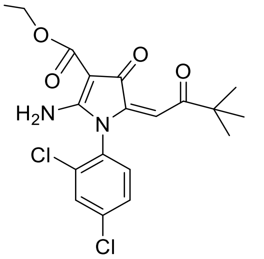 Molecular Weight: 411,28	$C_{19}H_{20}Cl_2N_2O_4$	CA-20	>100
26	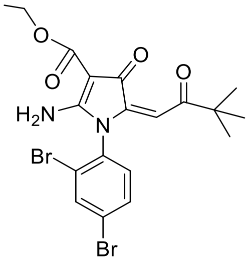 Molecular Weight: 500,19	$C_{19}H_{20}Br_2N_2O_4$	CA-28	>100
27	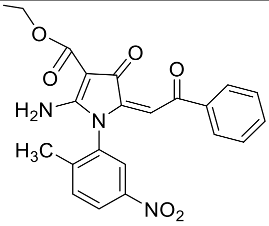 Molecular Weight: 421,41	$C_{22}H_{19}N_3O_6$	CA-33	54.1 ± 5.1
28	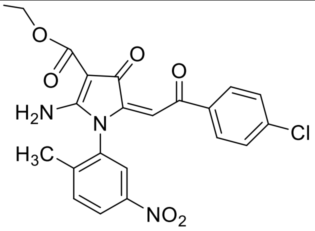 Molecular Weight: 455,85	$C_{22}H_{18}ClN_3O_6$	CA-59	6.2 ± 0.2
29	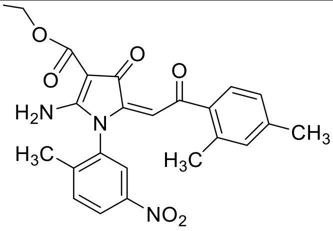 Molecular Weight: 449,46	$C_{24}H_{23}N_3O_6$	CA-60	33 ± 3.7
30	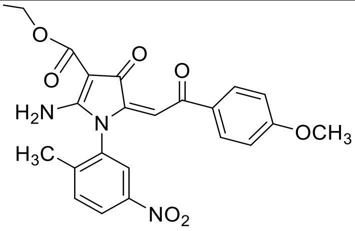 Molecular Weight: 451,44	$C_{23}H_{21}N_3O_7$	CA-68	80.7 ± 8.8
31	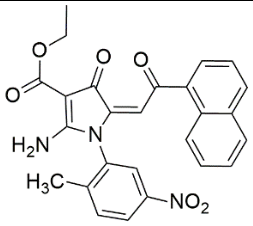 Molecular Weight: 471,47	$C_{26}H_{21}N_3O_6$	CA-90	30.3 ± 2.7

32	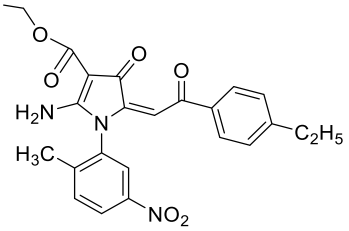 Molecular Weight: 449,46	$C_{24}H_{23}N_3O_6$	CA-166	5.1 ± 0.7
33	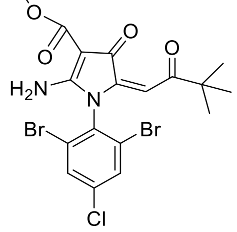 Molecular Weight: 534,63	$C_{19}H_{19}Br_2ClN_2O_4$	CA-174	83 ± 5.4
34	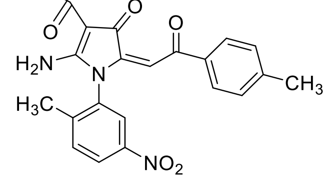 Molecular Weight: 435,44	$C_{23}H_{21}N_3O_6$	CA-208	38.2 ± 4.8
35	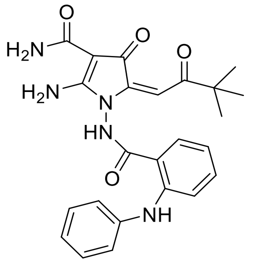 Molecular Weight: 447,50	$C_{24}H_{25}N_5O_4$	CA-84	4.8 ± 0.6
36	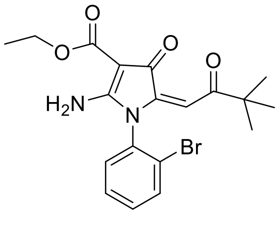 Molecular Weight: 421,29	$C_{19}H_{21}BrN_2O_4$	CA-26	>100
37	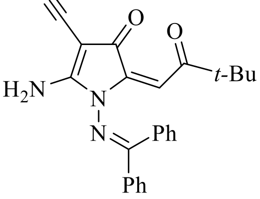 Molecular Weight: 398,47	$C_{24}H_{22}N_4O_2$	CA-23	34.9 ± 7.2

38	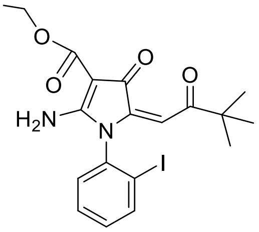<br>Molecular Weight: 468,29	$C_{19}H_{21}IN_2O_4$	CA-46	>100

Supplementary Table 2. *Structure-activity relationship (SAR)-based analysis of the features of the molecules in the dataset with observed activities.*

Group I

Core of structures

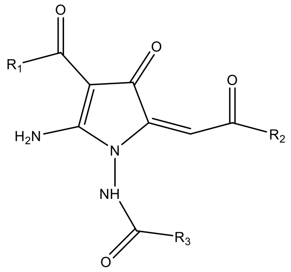

Compound	R1	R2	R3	IC_{50}
CA-61	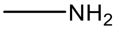	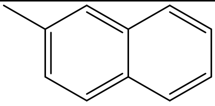	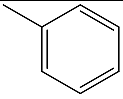	3.2
CA-1519I	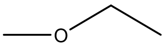	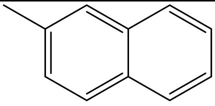	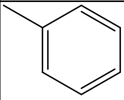	>100
CA-1573I	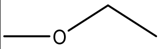	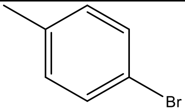	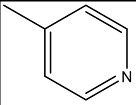	>100

Group II

Core of structures

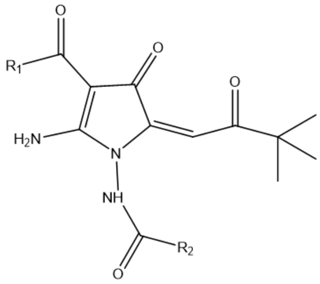

Compound	R1	R2	IC_{50}
CA-84	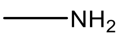	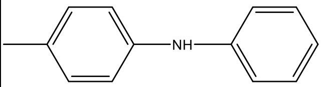	4.8
CA-1348I	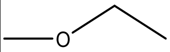	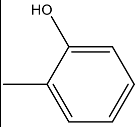	21.3
CA-610I	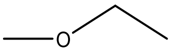	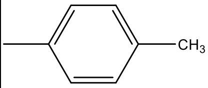	66

Group III

Core of structures

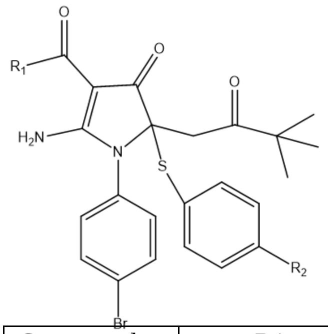

Compound	R1	R2	IC_{50}
CA-1489I	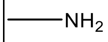	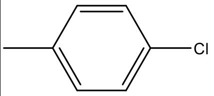	13
CA-1488I	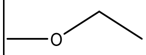	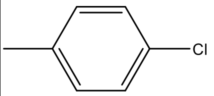	18.5
CA-1474I	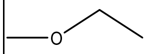	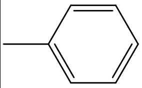	57.1

Croup IV

Core of structures

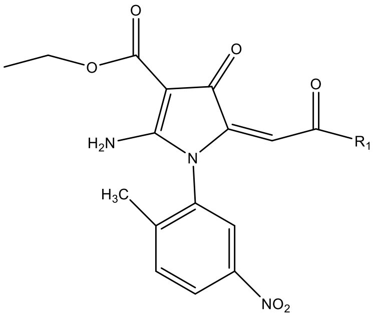

Compound	R1	IC_{50}
CA-166	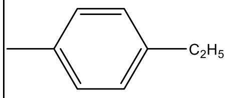	5.1
CA-59	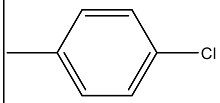	6.2
CA-90	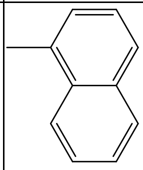	30.3
CA-60	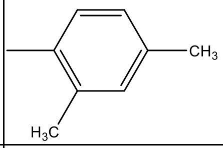	33.7
CA-208	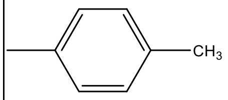	38.2
CA-33	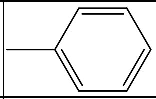	54.1
CA-68	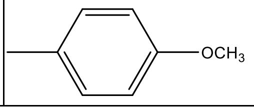	80.7
